# Virtual Reality–Based Intervention to Reduce Preoperative Anxiety in Adults Undergoing Elective Surgery: A Randomized Clinical Trial

**DOI:** 10.1001/jamanetworkopen.2023.40588

**Published:** 2023-10-31

**Authors:** Pak Lung Chiu, Huiyuan Li, Kevin Yi-Lwern Yap, Ka-man Carmen Lam, Pui-ling Renee Yip, Cho Lee Wong

**Affiliations:** 1 The Nethersole School of Nursing, Faculty of Medicine, The Chinese University of Hong Kong, Hong Kong SAR, China; 2 Department of Pharmacy, Singapore General Hospital, Singapore; 3 School of Psychology & Public Health, La Trobe University, Melbourne, Victoria, Australia; 4 Department of Anaesthesia and Operating Theatre Services, Hospital Authority New Territories West Cluster, Hong Kong SAR, China

## Abstract

**IMPORTANCE:**

Preoperative anxiety is common among adult patients undergoing elective surgery and is associated with negative outcomes. Virtual reality (VR)–based interventions have been considered simpler, safer, and more effective for reducing anxiety in patients undergoing surgery than conventional care.

**OBJECTIVE:**

To examine the effectiveness of a VR-based intervention with preoperative education in reducing preoperative anxiety among adult patients undergoing elective surgery.

**DESIGN, SETTING, AND PARTICIPANTS:**

An assessor-blinded prospective randomized clinical trial was conducted to recruit adult patients aged 18 years or older who were scheduled for their first elective surgery procedure under general anesthesia within the next 2 to 4 weeks at a preanesthesia assessment clinic in Hong Kong from July to December 2022.

**INTERVENTIONS:**

Participants were randomly assigned to either an intervention group (an 8-minute immersive 360° VR video tour in the operating theater via a head-mounted display console) or a control group (standard care).

**MAIN OUTCOMES AND MEASURES:**

The primary outcome of preoperative anxiety was measured using the Amsterdam Preoperative Anxiety and Information Scale (range, 6-30; higher scores indicate greater anxiety), and the secondary outcomes (ie, stress, preparedness, and pain) were assessed by Visual Analog Scale at 3 time points: baseline at beginning of clinical session (T0), at the end of the clinical session immediately after the intervention (T1), and before the surgery (T2). Pain, satisfaction levels, and postoperative length of stay were evaluated after the surgery (T3). Simulation sickness was assessed after the intervention by use of the Simulation Sickness Questionnaire. A generalized estimating equations model was applied to compare changes in outcomes over time.

**RESULTS:**

A total of 74 participants (mean [SD] age, 46.34 [14.52] years; 38 men [51.4%] and 36 women [48.6%]) were recruited and randomized to the control group (37 participants) and intervention group (37 participants). Compared with the control group, the VR-based intervention group showed significantly decreased preoperative anxiety at T1 (β, −5.46; 95% CI, −7.60 to −3.32; *P* < .001) and T2 (β, −5.57; 95% CI, −7.73 to −3.41; *P* < .001), lower stress at T1 (β, −10.68; 95% CI, −16.00 to −5.36; *P* < .001) and T2 (β, −5.16; 95% CI, −9.87 to −0.45; *P* = .03), and higher preparedness at T1 (β, 6.60; 95% CI, 0.97 to 12.19; *P* = .02). Satisfaction levels were significantly increased in the intervention group vs the control group (mean [SD] score, 81.35 [9.24] vs 65.28 [8.16]; difference, 16.07; 95% CI, 12.00 to 20.15; *P* < .001). No significant differences in pain and postoperative length of stay were found.

**CONCLUSIONS AND RELEVANCE:**

The findings of this study suggest that a VR-based intervention is a feasible and effective way to reduce preoperative anxiety in adult patients undergoing elective surgery. Given the promising results of this study, further study in the form of large-scale, multicenter, randomized clinical trials with broader implementation is warranted.

**TRIAL REGISTRATION:**

Chinese Clinical Trial Registry Identifier: ChiCTR2100051690

## Key Points

**Question:**

Compared with standard care, can a virtual reality–based intervention with preoperative education improve preoperative anxiety in adult patients undergoing elective surgery?

**Findings:**

This randomized clinical trial of 74 adult patients undergoing elective surgery found that a virtual reality–based intervention was effective in reducing preoperative anxiety.

**Meaning:**

The findings of this study suggest that virtual reality–based interventions can improve preoperative anxiety in adult patients undergoing elective surgery, but more randomized clinical trials with larger samples are needed to further confirm its effects.

## Teaser

This randomized clinical trial examines the effectiveness of a virtual reality (VR)–based intervention with preoperative education in reducing preoperative anxiety in adult patients undergoing elective surgery.

## Introduction

Surgical procedures are often associated with anxiety and stress.^1,2,3^ The incidence of preoperative anxiety varies widely, ranging from 11% in adult patients undergoing ear, nose, and throat surgical procedures to 80% in patients scheduled for neurosurgery.^4,5^ Although preoperative anxiety is considered a normal patient response, it can have negative physiological, emotional, and cognitive outcomes for patients.^6^ Preoperative anxiety can induce hypertension, increase heart rate and the risk of bleeding,^7^ and affect surgical outcomes and patient satisfaction.^8^ In addition, preoperative anxiety can increase the risk of postoperative complications, such as nausea, vomiting, respiratory distress, and heart attack,^9,10^ and is associated with high postoperative anxiety, postoperative pain, and prolonged length of stay,^11,12,13,14^ thereby affecting quality of life.^6,8^

Traditionally, pharmacological interventions have been used to manage preoperative anxiety,^15,16^ but they have drawbacks, such as transient irritation of mucosal membranes, postoperative nausea and vomiting, emergence of delirium, and prolonged length of stay.^17,18^ Nonpharmacological interventions, such as music therapy, distraction, and education, have also been incorporated into nursing care as preoperative interventions.^19,20,21,22^ A Cochrane systematic review^23^ of 26 studies has shown that music intervention can significantly decrease anxiety in patients compared with standard care. However, music interventions may require interventionists to receive adequate professional training to develop tailored music experiences, which may be limited in some health care settings. Distraction approach interventions, such as videos, multifaceted programs, interactive games, virtual reality (VR), and low sensory stimulation, have also been studied in the context of managing anxiety, particularly in children undergoing medical procedures and surgical procedures.^24,25,26,27^ However, the evidence for their effectiveness in the adult population remains unclear. Patient education through multimedia information, such as audiovisual videos, multimedia-supported education, and websites, has also been studied, but the results of previous systematic reviews^28,29^ have been inconsistent in terms of their effectiveness in reducing preoperative anxiety, pain, and length of hospital stay, compared with verbal or text-format education. Most importantly, these traditional and noninteractive interventions may not be able to meet the demands and expectations of patients in the current era of rapid technological development. Given the limitations of previously used nonpharmacological interventions, the development of novel advanced technologies, such as VR, provides promising directions for managing preoperative anxiety.

VR allows humans to visualize, manipulate, and interact with a computer-generated, simulated 3-dimensional image or environment through human-computer interaction.^30,31^ In an immersive VR environment, a head-mounted display (HMD) with a wide field of view projects the simulated image to the user’s eyes, along with a body-tracking motion sensor system based on a built-in gyroscope.^32^ The simulated virtual scene, complete with visual imagery and sounds, can induce the user’s immersion and interaction with a virtual world.^33^ As VR technology continues to evolve into smaller and more powerful systems, wearable and lightweight VR systems have become usable, accessible, and affordable in health care settings.^34,35^

The application of VR for managing preoperative anxiety has shown more promising effects than traditional means. Four previous randomized clinical trials (RCTs)^36,37,38,39^ using different VR-based interventions (eg, VR tour of an operating theater, VR exposure therapy, and VR game) reported significant reductions in preoperative anxiety in children undergoing elective surgery. In addition, Ryu et al^37^ found that induction compliance and stressful behaviors of children, as well as parental satisfaction, were also increased with use of a VR intervention. Only one study^32^ indicated that providing a 15-minute, VR computer-generated, child-friendly operating theater environment, without usual care, was associated with significantly less rescue analgesia, despite no beneficial effects on anxiety. Studies on managing preoperative anxiety with VR for adults have also been identified. However, the subgroup analysis in a systematic review involving 5 RCTs^40,41,42,43,44^ in adult patients did not observe significantly lower preoperative anxiety.^45^ Some limitations exist in these RCTs. First, these studies focused on specific surgical procedures, which may limit the generalizability of the findings to diverse types of operations. Second, different VR techniques developed within specific surgical contexts may not be well suited to other surgical procedures. Although 2 additional RCTs^46,47^ showed positive results in reducing anxiety and stress in adult patients scheduled for specific surgical procedures, there is a paucity of evidence to develop a standard VR-based intervention for adult patients undergoing different elective surgical procedures and to investigate its effects on reducing preoperative anxiety in this population. Hence, the current study aimed to evaluate the VR-based intervention to reduce preoperative anxiety in adult patients undergoing elective surgery.

## Methods

This was a prospective, single-blind RCT conducted from July to December 2022. However, owing to the COVID-19 pandemic, the study was suspended after December 2022. The protocol is provided in Supplement 1. This study adhered to the Consolidated Standards of Reporting Trials (CONSORT) reporting guidelines. The study complied with the Code of Ethics and guidelines set forth by the Declaration of Helsinki.^48^ It was approved by the Hospital Authority New Territory West Cluster Research Ethics Committee and the Joint Chinese University of Hong Kong-New Territories East Cluster Clinical Research Ethics Committee. Written informed consent was obtained from each participant before data collection. The confidentiality and anonymity of the participants’ information were ensured, and their right of withdrawal without recrimination or prejudice was highlighted.

### Participants and Setting

The study was conducted at the preanesthesia assessment clinic of a public general district hospital in Hong Kong. Patients attending the preanesthesia assessment clinic were invited to participate in the study if they met the inclusion criteria, which included (1) being at least 18 years old, (2) scheduled for their first elective surgery procedure under general anesthesia within the next 2 to 4 weeks, (4) spoke Cantonese, and (5) were grade I or II of American Society of Anesthesiologists (ASA) Physical Status Classification System. Patients were excluded if they (1) were undergoing an emergency procedure without prior preoperative assessment, (2) were cognitively impaired or unable to consent, (3) had a history of any psychological disorder, and (4) had a history of vestibular dysfunction or motion sickness, had alcohol or substance abuse, or had Parkinson disease, multiple sclerosis, or muscular dystrophy.

### Sample Size

According to a meta-analysis^45^ of 10 RCTs examining the effects of VR-based interventions on preoperative anxiety through different scales in measurement, a sample size of at least 45 per group, with a total of 90 participants, was deemed sufficient to detect an effect size of 0.64 between the intervention and control groups, with a statistical power of 80% and an α of 5%, allowing for a 10% attrition rate at time 3. However, owing to the COVID-19 pandemic, several elective surgery sessions were canceled or deferred in the study period, and we were not able to recruit 90 participants.

### Recruitment Procedure

Consecutive sampling was used to recruit participants who attended the preanesthetic assessment clinic for scheduled elective surgery under general anesthesia. Participants were identified through a medical record review and were approached for eligibility by an independent research assistant. The list of interested participants was recorded and sent to the researcher for further contact. The researcher explained the study details to potential participants. After obtaining written consent from the patient, an independent outcomes assessor blinded to the group allocation collected baseline data (T0).

### Randomization and Blinding

The randomization procedure was performed by a research assistant who was not involved in the recruitment, enrollment, and treatment process. Participants were randomly assigned at a 1:1 ratio to either the VR-based intervention group or the control group using block randomization (block size, 4). The research assistant who performed randomization prepared opaque, sealed envelopes numbered sequentially to conceal the allocation sequences and delivered them to participants to inform allocation results.

Because of the nature of the intervention, only the outcome assessor was blinded to the group allocation. The participants and interventionists could not be blinded. Nevertheless, the participants were instructed not to reveal their group allocation to the outcome assessor at the time of outcome assessment and were asked not to share details about the intervention with others.

### Control Group

Participants in the control group received standard care in the preanesthetic assessment clinic. Routine preoperative information was provided to participants in the control group by the perioperative nurses in the clinic.

### Intervention Group

In addition to standard care, participants in the intervention group received a VR-based intervention. The VR-based intervention consisted of a VR video created with a high-quality 360° camera at a resolution of 5760 × 2880 pixels a 30 frames-per-second format with 360° directional sound. The VR video included a virtual tour simulating the entire journey of the perioperative process, from entering the operating theater to being transferred to the recovery area, including introduction, reception, induction, operating theater, postanesthesia care unit, and ending (eFigure 1 in Supplement 2). This provided the participants with immersive first-person exposure and experience of the operating theater and surgery process.

The participants in the intervention group were provided with a VR console (Oculus Quest 2; Meta) in a separate room within the clinic during their visit to watch the VR video. Supporting staff assisted the participants in using the device whenever necessary. The console is an all-in-one, high-quality, standalone HMD VR console that operates without the need for computer or device attachment. It has a screen resolution of 1832 × 1920 pixels, a refresh rate of 90 Hz, integrated speakers and microphone, 2 to 3 hours of battery life, and adjustable interpupillary distance with 3 settings for 58 mm, 63 mm, and 68 mm. An additional accessory, the Elite Strap, was applied to the console to enhance its wearability, head support, and comfort. After consulting with the hospital’s senior nursing officer of infection control unit, infection control measures were taken by applying disposable eye mask covers and using alcohol wipes for disinfection after use. Screen captures of the VR video are shown in eFigure 1 in Supplement 2.

### Intervention Fidelity

Five nursing staff members at the preanesthetics assessment clinic received 30 minutes of training on how to operate the HMD console. The training included instructions on setting up the devices, adjusting the interpupillary distance on the HMD consoles, troubleshooting potential issues, and separating participants into control and intervention groups. A standardized procedure for implementing the VR-based intervention was developed to ensure the nurses’ adherence to the study protocol.

### Outcome Measures

#### Primary Outcome: Preoperative Anxiety

Preoperative anxiety was measured using the Amsterdam Preoperative Anxiety and Information Scale (APAIS).^49^ The scale consists of 6 items rated on a 5-point Likert scale divided into 2 subscales: the anxiety scale and the need-for-information scale, with total score ranging from 6 to 30. Higher scores indicate higher anxiety levels or greater information needs. This study used the translated and validated Chinese version of the APAIS,^50^ with Cronbach α of 0.862 and 0.830 for the anxiety and the need-for-information subscales, respectively.

#### Secondary Outcomes

The secondary outcomes included pain at rest (measured using the Visual Analog Scale [VAS] to assess the subjective pain^51^), stress (measured using the VAS), preparedness (measured using the VAS), simulation sickness (measured using the Simulation Sickness Questionnaire^52,53^), satisfaction (measured using the VAS), and postoperative length of stay (the time from the end of surgery and anesthesia and transfer to the postanesthetic care unit) to discharge from the surgical in-patient episode). The eAppendix in Supplement 2 provides detailed descriptions of each outcome measure.

### Data Collection

The participants’ sociodemographic (age, sex, marital status, living arrangement [living alone or not], and educational level) and clinical (current medication regimen and medical history) data were collected by the blinded outcome assessor at the beginning of the preanesthetic assessment clinic session (T0, baseline), close to the end of the clinical session immediately after the intervention (T1, postintervention), before transference to the operation theater (at ward) or after admission to the operation theater (at the reception area) (T2), and between 24 and 48 hours after the surgery (T3). If any psychosomatic discomfort reaction was observed in participants, the intervention would be terminated, and corresponding support would be provided to help them relax. Any adverse events were reported to the institutional review board of the Chinese University of Hong Kong.

### Statistical Analysis

SPSS statistical software version 29.0 (IBM) was used for data analysis. The demographic characteristics and baseline outcome measures were summarized by frequency, percentage, mean (SD), and range. Normal Q-Q plots of skewness and kurtosis of data were used to assess continuous variables’ normality. Normality was further evaluated by using the Shapiro-Wilk test to determine the skewness and kurtosis of the data sets. The χ^2^ test or Fisher exact test was used for categorical variables to compare the homogeneity of characteristics between the interventional and control groups, whereas the independent samples *t* test or the Mann-Whitney *U* test was used for continuous variables. The outcome comparison was adjusted by those significant variables as confounding factors. The independent sample *t* test, Mann-Whitney *U* test, χ^2^ test, and Fisher exact test were used to compare the preoperative anxiety, pain, stress, preparedness, satisfaction, and lengths of stay, and the Wilcoxon signed-rank test was used for simulation sickness before and after the intervention. The univariable generalized estimating equations (GEE) model using unstructured correlation structure was used to evaluate the effect of the intervention by comparing the changes in the outcome variables between the intervention and the control groups over time.^54^ An intention-to-treat analysis was adopted in this study. A cutoff of 11 on the APAIS score was recommended for screening and diagnostic purposes on the basis of comparison to the State-Trail Anxiety Inventory. However, GEE allows us to model the association of the intervention with anxiety scores over all 3 study time points (T0, T1, and T2), accounting for within-participant correlation of repeated measures. This maximizes statistical power compared with separate analyses at each time point.^49^ Two-sided *P *<* *.05 was regarded as statistically significant.

## Results

### Characteristics of Participants

A total of 157 participants were assessed for eligibility. Of these, 74 participants (47.1%) agreed to participate and provided written consent (mean [SD] age, 46.34 [14.52] years; 38 men [51.4%] and 36 women [48.6%]); 37 participants each were randomized to the intervention and control groups. All participants in both the intervention and control groups completed the interventions and follow-up assessments at the end of the clinic session, except for 1 participant in the control group who was discharged before data collection. No major adverse events were reported. Therefore, the overall attrition rate at T1 and T2 was 0%, and the attrition rate was 0% in the intervention group and 2.7% in the control group. The Figure shows the study flow. Table 1 summarizes the demographic characteristics of the 74 participants.

**Figure.**
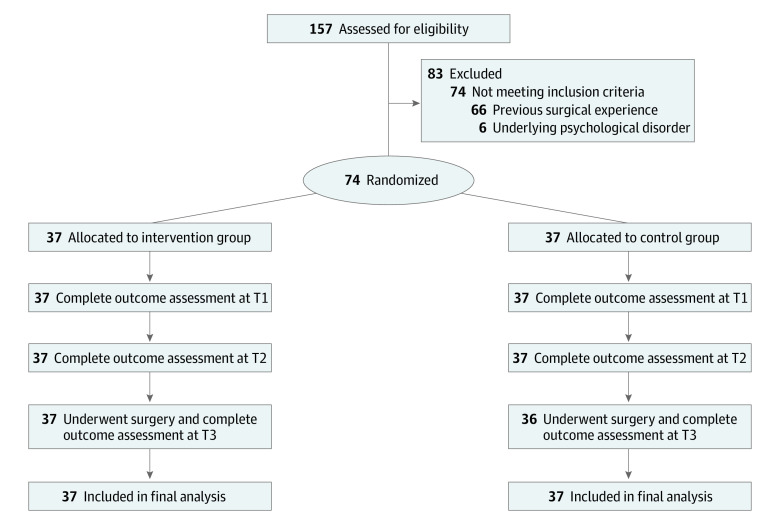
Participant Enrollment Flowchart T1 refers to the end of the clinical session immediately after the intervention, T2 refers to the time before transference to the operation theater or after admission to the operation theater and before the surgery, and T3 refers to 24 to 48 hours after the surgery.

**Table 1.** Sociodemographic Characteristics of the Participants

Characteristic	Participants, No. (%) (N = 74)	
	Control group (n = 37)	Intervention group (n = 37)
Age, mean (SD) [range], y	45.35 (15.94) [18.00-69.00]	47.32 (13.09) [22.00-69.00]
Sex		
Male	18 (48.6)	20 (54.1)
Female	19 (51.4)	17 (45.9)
Marital status		
Not married (single, widowed, or divorced)	19 (51.4)	14 (37.8)
Married	18 (48.6)	23 (62.2)
Educational level		
Primary or below	9 (24.3)	23 (62.2)
Secondary school	13 (35.1)	17 (45.9)
University or above	15 (40.5)	11 (29.7)
Living arrangement		
Living alone	15 (40.5)	12 (32.4)
Living with others	22 (59.5)	25 (67.6)
Employment status		
Employed	16 (43.2)	22 (59.5)
Not at work (unemployed, between jobs, housekeeper, student, or retired)	21 (56.8)	15 (40.5)
American Society of Anesthesiologists grade I	37 (100.0)	37 (100.0)
Experience of admission to hospital		
No	21 (56.8)	14 (37.8)
Yes	16 (43.2)	23 (62.2)
Type of operation		
General surgery	5 (13.5)	5 (13.5)
Functional endoscopic sinus surgery	9 (24.3)	13 (35.1)
Arthroscopic surgery	19 (51.4)	14 (37.8)
Transurethral resection surgery	4 (10.8)	5 (13.5)

### Changes in Health Outcomes Before and After Intervention

Table 2 summarizes the mean scores of the preoperative anxiety and secondary outcomes at baseline and after the intervention. No significant differences were found in these outcomes between the intervention and control groups at baseline. No simulation sickness was reported at baseline for participants in the intervention group. Only 1 participant reported experiencing dizziness with eyes open after the intervention as measured by Simulation Sickness Questionnaire, but the feeling disappeared immediately after removing the HMD console.

**Table 2.** Comparison of Outcomes Scores Between Study Groups at 3 Time Points^a^

Outcome variables	Score, mean (SD)			
	Baseline	T1	T2	T3
Preoperative anxiety^b^				
Control group	23.03 (3.09)	20.35 (4.86)	20.59 (4.82)	NA
Intervention group	23.92 (2.19)	15.78 (4.51)	15.92 (4.67)	NA
Anxiety subscale				
Control group	15.35 (2.16)	13.46 (3.44)	13.89 (3.86)	NA
Intervention group	15.59 (1.89)	10.00 (3.39)	10.32 (3.63)	NA
Need for information subscale				
Control group	8.00 (1.03)	6.89 (1.87)	6.97 (1.88)	NA
Intervention group	8.32 (1.08)	5.78 (1.83)	5.86 (1.95)	NA
Stress^b^				
Control group	60.95 (12.70)	52.41 (14.12)	67.16 (11.91)	NA
Intervention group	58.65 (13.91)	39.43 (18.26)	59.70 (17.10)	NA
Preparedness^b^				
Control group	23.62 (5.53)	41.59 (5.78)	39.76 (6.04)	NA
Intervention group	23.70 (6.27)	48.27 (17.15)	42.57 (16.51)	NA
Pain^b^				
Control group	0.95 (4.38)	1.65 (5.80)	26.86 (14.19)	63.81 (17.91)
Intervention group	1.27 (4.52)	1.92 (5.72)	26.54 (15.95)	61.49 (16.78)
Satisfaction^b^				
Control group	NA	NA	NA	65.28 (8.16)
Intervention group	NA	NA	NA	81.35 (9.24)
Postoperative length of stay				
Control group	NA	NA	NA	55 (10-169)^c^
Intervention group	NA	NA	NA	54 (27-128)^c^
Simulation sickness				
Control group	NA	NA	NA	NA
Intervention group	0	1.41 (8.56)	NA	NA
Nausea subscale				
Control group	NA	NA	NA	NA
Intervention group	0	0	NA	NA
Oculomotor subscale				
Control group	NA	NA	NA	NA
Intervention group	0	0	NA	NA
Disorientation subscale				
Control group	NA	NA	NA	NA
Intervention group	0	0.38 (2.29)	NA	NA

Abbreviation: NA, not applicable.

^a^ T1 is after the intervention. T2 is the time before transference to the operation theater (at ward) or after admission to operation theater (at the reception area). T3 is between 24 and 48 hours after the surgery.

^b^ Data between groups were compared with independent *t* test.

^c^ Data between groups were compared with Mann-Whitney *U* test. Data are median (range) days.

### Effects of VR-Based Intervention on Preoperative Anxiety

Table 3 presents the results of the GEE model for the outcomes between study groups across time. The GEE analysis showed that the interaction effects were significantly different for the total score of preoperative anxiety at T1 (β, −5.46; 95% CI, −7.60 to −3.32; *P* < .001) and at T2 (β, −5.57; 95% CI, −7.73 to −3.41; *P* < .001) and for the anxiety subscore and need-for-information subscore at T1 and T2 (eFigure 2 in Supplement 2).

**Table 3.** Changes in the Outcomes Between Study Groups Across Time Using Generalized Estimating Equation Model (N = 74)

Outcomes	Group^a^		T1^a^		Group*T1^a^		T2^a^		Group*T2^a^	
	β (95% CI)^b^	*P* value	β (95% CI)^b^	*P* value	β (95% CI)^b^	*P* value	β (95% CI)^b^	*P* value	β (95% CI)^b^	*P* value
Primary outcome, preoperative anxiety	0.89 (−0.31 to 2.10)	.15	−2.67 (−4.28 to −1.10)	<.001	−5.46 (−7.60 to −3.32)	<.001	−2.43 (−4.00 to −0.87)	.002	−5.57 (−7.73 to −3.41)	<.001
Anxiety subscale	0.24 (−0.67 to 1.16)	.60	−1.89 (−2.94 to −0.85)	<.001	−3.70 (−5.21 to −2.20)	<.001	−1.46 (−2.67 to −0.25)	.02	−3.81 (−5.49 to −2.14)	<.001
Need for information subscale	0.32 (−0.15 to 0.80)	.18	−1.11 (−1.63 to −0.59)	<.001	−1.43 (−2.17 to −0.70)	<.001	−1.03 (−1.55 to −0.51)	<.001	−1.43 (−2.22 to −0.65)	<.001
Secondary outcomes										
Stress level	−2.30 (−8.29 to 3.69)	.45	−8.85 (−10.37 to −6.71)	<.001	−10.68 (−16.00 to −5.36)	<.001	6.22 (4.93 to 7.51)	<.001	−5.16 (−9.87 to −0.45)	.03
Preparedness	0.08 (−2.58 to 2.74)	.95	17.97 (16.46 to 19.49)	<.001	6.60 (0.97 to 12.19)	.02	16.14 (14.64 to 17.64)	<.001	2.73 (−2.70 to 8.13)	.32
Pain^c^	0.32 (−1.68 to 2.33)	.75	0.70 (−0.16 to 1.57)	.11	−0.05 (−1.25, 1.14)	.94	25.91 (21.03 to 30.81)	<.001	−0.65 (−7.99 to 6.69)	.86

^a^ Group refers to the group differences at baseline between the intervention and control groups. Group*T1 refers to additional score changes of the variables in the intervention group compared with those in the control group at T1. Group*T2 refers to additional score changes of the variables in the intervention group compared with those in the control group at T2. T1 is close to the end of the clinical session (postintervention), and T2 is before transference to the operation theater (at ward) or after admission to operation theater (at the reception area).

^b^ β refers to the group-by-time interaction terms and represents the difference in mean changes in an outcome at a specific time point with respect to the baseline (T0) between the intervention and control groups (mean change in intervention group minus mean change in control group).

^c^ Changes in pain scores were also examined at T3 (after operation) between groups (T3, 62.86; 95% CI, 57.36 to 68.37; *P* < .001; group*T3, −2.65; 95% CI, 10.15 to 4.85; *P* = .49).

### Effects of VR-Based Intervention on Secondary Outcomes

Participants in the VR-based intervention group reported significant decreases in stress levels compared with the control group at T1 (β, −10.68; 95% CI, −16.00 to −5.36; *P* < .001) and T2 (β, −5.16; 95% CI, −9.87 to −0.45; *P* = .03) (Table 3 and eFigure 3 in Supplement 2). Significant increases were observed in the scores of preparedness between the groups at T1 (β, 6.60; 95% CI, 0.97 to 12.19; *P* = .02), but no statistically significant interaction effects were detected at T2 (Table 3 and eFigure 4 in Supplement 2). In addition, no significant changes were observed in the pain level scores at the 3 time points (eFigure 5 in Supplement 2). The satisfaction level after surgical operations (T3) was significantly higher in the intervention group compared with the control group (mean [SD] score, 81.35 [9.24] vs 65.28 [8.16]; difference, 16.07; 95% CI, 12.00 to 20.15; *P* < .001) (Table 2 and eFigure 6 in Supplement 2). Regarding length of stay, the intervention group reported a shorter postoperative length of stay (median, 54 days; range, 27-128 days) than the control group (median, 55 days; range, 10-169 days). However, no significant differences were noted (Table 2 and eFigure 7 in Supplement 2). No inferential analysis was conducted because only 1 participant reported simulation sickness after the intervention.

## Discussion

This RCT is one of the few to investigate the effects of a VR-based intervention in reducing preoperative anxiety among adult patients undergoing elective surgery in a preanesthetic assessment clinic setting. The results provide promising evidence that the VR-based intervention can effectively improve preoperative anxiety, stress, preparedness, and pain in adult patients undergoing elective surgery.

A significant reduction in preoperative anxiety was observed by adopting the VR-based intervention in a preanesthetic assessment clinic. These results align with those of prior related studies.^40,44^ VR exposure allows participants to experience a computer-generated surgical environment and procedures, helping desensitize them to anxiety-provoking aspects of the real experience. This exposure in a controlled setting can help reduce anticipatory fear and distress.^55^ The immersive nature of VR helps patients feel as though they are in a realistic environment, enhancing its potential impact. Patients learn what the operating room looks like and hear the sounds from equipment in the operating theater in a safe yet controlled environment. The VR-based intervention also allows patients to explore the operating theater at their own pace.

Another possible reason for the significant reduction in preoperative anxiety among participants in the VR-based group could be the information guidance they received about their anesthesia and surgical procedures. Previous studies have shown that providing preoperative information to patients, including the journey to the operating theater for surgery, effectively decreases preoperative anxiety.^36,38,56,57^ When participants have a detailed understanding of what to expect before, during, and after their operation, distressing uncertainty and preoperative anxiety can be minimized. VR technology offers an advanced method of providing this information in a safer, more controlled, and cost-effective manner.

The results of this study revealed that participants in the VR-based intervention group experienced significantly less stress and better preparedness compared with the control group. These findings are consistent with those of similar studies.^40,44,46,47^ In this study, patients were provided with a preoperative virtual tour using an immersive 360° VR video in audiovisual format. This type of video is more realistic, allowing patients to experience the actual surgical journey in a personalized, safer, and more controlled environment than virtual environment with avatars.

The participants’ satisfaction level toward the preoperative services was significantly higher in the intervention group than in the control group. Previous studies have also found positive results when applying VR interventions before and during surgery^44^ and intraoperatively.^40^ VR gave participants a sense of control over the environment and insight into what to expect from a real-life situation. With prior exposure to the surgical operation, patients perceived VR as a helpful way to prepare for the unfamiliar and anxiety-provoking experience of surgery, which may promote better satisfaction.

This study found no significant effects of the VR-based intervention on pain levels. These results differ from those of a previous meta-analysis.^58^ Nonetheless, the potential of adopting VR-based interventions in the intraoperative and postoperative periods warrants further exploration.

Similar to previous studies,^40,44,46,47^ this study found no significant effects of the VR-based intervention in reducing patients’ length of postoperative hospitalization. One possible explanation may be that the requirement for medical and nursing care after an operation may vary among different types of surgical procedures, surgeons, and units of care, and the limited sample size could not provide a specific subgroup analysis of the postoperative length of stay.

### Strengths and Limitations

There are several strengths of this study. First, it adopted an RCT design, which is considered the criterion standard for evaluating the cause-and-effect relationships between interventions and outcomes. Second, to our knowledge, it was the first study to investigate VR-based interventions in a preanesthetic assessment clinic setting, providing valuable insights into its potential as a nonpharmacological approach to reducing preoperative anxiety and improving outcomes in adult patients undergoing elective surgery.

There are also some limitations to be considered. First, because the 0.64 effect estimator corresponded to the standardized mean difference for different scales to measure preoperative anxiety in the previous meta-analysis and although it was adopted for sample size calculation, this could not precisely imply that a score reduction of at least 0.64 on the APAIS indicates a relevant reduction in anxiety. Furthermore, the study was conducted during the COVID-19 pandemic, and up to 50% of elective surgical procedures had been canceled or deferred. Therefore, only 74 participants were recruited, and the power of the intervention may be limited. Second, this study was conducted in a single preanesthetic assessment clinic, and the standard of care and provision of service may vary among different clinics. Also, the recruitment of mostly young participants with ASA grade I status for relatively low-risk, short duration, or less-pain-induced surgical procedures may limit the generalizability of the findings to patients aged more than 80 and 90 years or patients with higher ASA risk. Patients in different assessment clinics for different types of surgical procedures should be investigated in future studies. Third, owing to the nature of the intervention, the allocation of intervention was unable to be concealed from the participants, and those in the intervention group may have been more susceptible to performance bias by subjectively providing more positive self-reports that aligned with the perceived goals of their assigned intervention during the outcome measurement.^59^ Fourth, as the underprivileged population, such as older and less educated people, was more vulnerable to an increase in stress levels and anxiety^60^ during the COVID-19 pandemic, the influence of potential unmeasured confounding phenomena, such as the pandemic, on preoperative anxiety levels remains unknown and could have affected the study results. Fifth, because there is no well-established tool in this specific setting to evaluate stress, preparedness, and satisfaction, the exploratory outcomes using VAS should be interpreted with precaution.

## Conclusions

In conclusion, the findings of this trial indicate that VR-based interventions can be effective in reducing preoperative anxiety and stress levels, as well as promoting preparedness and service satisfaction in adult patients undergoing elective surgery. However, more RCTs with larger samples are needed to further confirm their effects and improve external validity. Findings from this study support research to explore the role of VR technology in other nursing care and health service settings to help manage preoperative anxiety.

## Notes

Supplement 1.
Trial Protocol and Statistical Analysis Plan

Supplement 2. **eFigure 1.** Screen Captures of VR-Based Intervention Throughout the Entire Perioperative Process **eFigure 2.** Changes of Preoperative Anxiety Scores Across Three Time Points **eFigure 3.** Changes of Stress Level Across Three Time Points **eFigure 4.** Changes of Preparedness Across Three Time Points **eFigure 5.** Changes of Pain Level Across Four Time Points **eFigure 6.** Graphical Comparison of the Satisfaction of the Study Groups at T3 **eFigure 7.** Graphical Comparison of Postoperative Length of Stay Between Study Groups **eAppendix.** A Detailed Description of Each Measure Used in This Trial
eReferences

Supplement 3.
Data Sharing Statement
