# Supplementary material for: Virtual Reality–Based Intervention to Reduce Preoperative Anxiety in Adults Undergoing Elective Surgery: A Randomized Clinical Trial
Source: JAMA Netw Open. 2023 Oct 31;6(10):e2340588. doi: 10.1001/jamanetworkopen.2023.40588 (PMC10618840; doi:10.1001/jamanetworkopen.2023.40588)
Supplement: Supplement 1. — Trial Protocol and Statistical Analysis Plan [file jamanetwopen-e2340588-s001.pdf]

Effects of virtual reality based intervention on reducing preoperative anxiety among adult patients undergoing elective surgery: a randomized controlled trial

Version1, 19 August 2021

Introduction

Surgery is a stressful experience to most of the patients and commonly associated with anxiety (Calvin &Lane, 1999; Mitchell, 2003; Wiens, 1998), in which, anxiety, induced by autonomic and somatic responses, is an unpleasant feeling of unease, discomfort, apprehension, fearful concern, tension and vigilance (Bedaso &Ayalew, 2019; Shri, 2010). Depending on the assessment method, incidence of preoperative anxiety was estimated ranges from 11% to 80% in adult (Kain &Maranets, 2000). It could be induced by concerns related to general health and uncertainty regarding the outcome, type of surgery and anesthesia, pain and discomfort after the surgery, feeling of helplessness, loss of independence and fear of mortality (Caumo et al., 2001), although it often accepted negligently as a normal response (Kain &Maranets, 2000).

In responding to anxiety state as a personality feature in certain situation for handing a threat or dangers, stress could be provoked by the triggering of hypothalamic-pituitary-adrenal axis and sympathetic nervous system, in which hormones, tissue plasminogen activator and catecholamines would be released to activate the fight-or-flight response (Hoirisch-Clapauch, 2018; Steimer, 2002). Hence, preoperative anxiety would not only induce hypertension and increased heart rate but also disturb the balance between hypercoagulation and hyperfibrinolysis which increases the chance of bleeding (Hoirisch-Clapauch, 2018). The fluctuation of autonomic response is also associated with the anesthetic requirement that increase the incidence of complications such as nausea, vomiting, respiratory distress and heart attack (Celik &Edipoglu, 2018; Perks et al., 2009). A wide range of negative consequences, including physiological, emotional and cognitive effects (Wong et al., 2010), could be resulted and affected the outcomes of the surgery (Bedaso &Ayalew, 2019). The increase of adrenaline,

norepinephrine and plasma cortisol caused by the stress would suppress the immunological response and increase risks of postoperative complications to the patients (Almalki et al., 2017; Ramos et al., 2008). For instance, it is recognized that the preoperative anxiety impose direct cost and complications in the orthopaedic surgery (Rasouli et al., 2016).

High incidence of preoperative anxiety is significantly associated with negative impact on patient, organization and healthcare system. Therefore, a substantial number of studies have been conducted to identify effective pharmacological and non-pharmacological interventions to address preoperative anxiety.

Challenges in Managing Preoperative Anxiety

Traditionally, pharmacological interventions using sedatives and anti-anxiety drugs, include intravenous midazolam, ketamine, dexmedetomidine, transmucosal fentanyl, and oral clonidine (Bauer et al., 2004; McCann &Kain, 2001), can provide rapid and reliable effects in managing preoperative anxiety. Nevertheless, the drawbacks of anxiolytic including painful cannulation, transient irritates the mucosal membranes, postoperative nausea and vomiting, emergence delirium, excessive salivation, postoperative sedation and prolonged length of stay unfortunately jeopardize the outcomes of cares (Filatov et al., 2000; Kumar Kar &Ganguly, 2015; McCann &Kain, 2001).

Balancing the risk and benefit of the interventions, non-pharmacological interventions, such as clown therapy (Messina et al., 2014), music therapy and music medicine interventions (Bradt et al., 2013), information leaflet (Bellew et al., 2002), and audio-visual video (Ayral et al., 2002), or a combination of them (Lee et al., 2004), are a more preferable option to manage preoperative anxiety in general setting and is recommended to incorporate as a standard preoperative nursing intervention (Croke, 2020). However, some limitations might hinder the applicability of aforementioned interventions.

Music therapy and music medicine interventions, as suggested in a Cochrane review (Bradt et al., 2013), would lower the state anxiety, while trained music therapists to individualize a tailored music experience were required to provide music interventions. Hence, proper music interventions training for providing personalized experience might encumber nurses and other healthcare professions during implementation (Bradt et al., 2013).

Providing information through patient education, such as information leaflet (Bellew et al., 2002) and audiovisual video (Ayral et al., 2002), were also suggested in studies, which expected to effectively reduce preoperative anxiety, as concluded in two systematic reviews (Alanazi, 2014; Hounsome et al., 2017) that the use of audiovisual, visual only, multimedia-supported education, websites, verbal education and information leaflets demonstrated a significantly reduction of preoperative anxiety. However, textual based information leaflet may not be suitable for patients with limited health literacy (Guo, 2015).

Preoperative orientation tour also demonstrate positive effect on anxiety, as shown in at least two RCTs (Battah et al., 2021; Niknejad et al., 2019), while physical tour would be a great challenge in term of resources in manpower and venue as well as infection control concerns.

Virtual Reality Based Interventions

With the emerging development of technology, virtual reality (VR) based intervention has been widely incorporated into preoperative care as a newfangled direction and offers enormous opportunities to effectively and dexterously address preoperative anxiety, which is considered as easier, safer, and more effective than conventional methods (Eijlers, Utens, et al., 2019).

Individuals would be allowed to visualize, manipulate and interact with computer-generated simulated three-dimensional image or environment through human-computer

interaction through virtual reality technology (Freina &Ott, 2015; Rizzo &Koenig, 2017). A person can immerge, through different technological approaches, into an imaginary but convincing virtual world by engaging images, sound, and other sensory elements from a synthetic computerized environment (Greengard, 2019), namely non-immersive and immersive VR.

Non-immersive virtual reality, as in the earlier years, refers to the synthetic three-dimensional environment delivered on a standard flat-screen television or computer monitor with no obsoletion of the outside world (Rizzo &Koenig, 2017) with least interactive implementation of virtual reality technology (Shahrbanian et al., 2012). The technology has been commonly adopted by computer games and videogame consoles in which the user could interact with and navigate within the computer environment through a keyboard and a mouse, a joystick or a specialized interface device (Rizzo &Koenig, 2017), such as accelerometer and gyroscope equipped Joy-Con adopted by Nintendo Switch (Nintendo, 2019).

Alternatively, in an immersive virtual reality environment, the simulated image or environment shall projected to the users' eye through advanced head-mounted displays with wide field of view and body-tracking motion sensor system via build-in gyroscope (Eijlers et al., 2019a). The technology allows users operate in a simulated computer generated environment which could response to head or body movement. Immersive often refers to occlusion of user's visual view of the outside world with engaging user in a virtual world that responding to motion tracking information to a computerized system that creating sensory stimuli in nearly real-time to the activities (Rizzo &Koenig, 2017). The simulated virtual scene could induce illusion of a user by visual imagery and sounds to be immersed in and interact with a virtual world (Foronda et al., 2017).

Whilst application of virtual reality for managing preoperative anxiety has advantages and promising effect over traditional means, many standalone or smartphone-based virtual

reality devices such as Oculus Go, Samsung Gear VR and Google Cardboard are currently available in the market (Facebook, 2020; Google, 2020). With the evolution of virtual reality technology to smaller, more compact but yet powerful system, wearable and lightweight virtual reality system becomes accessible, affordable, easily implemented and deployed in most of the general clinical setting by nursing team and other healthcare profession (Greengard, 2019; Parsons et al., 2009).

Although VR technology may provide notable opportunity for managing preoperative anxiety in a newfangled approach, previous studies encompassed a variety of population, interventions and approaches. Although a meta-analysis of randomized controlled trials of investigating the effect of VR on preoperative anxiety and concluded that the positive effect for adult population (Koo et al., 2020), there were no local study conducted in the local setting and in the Chinese population. Thus, the evaluation of current evidence is imperative to adopt this novel intervention to address this prevailing problem in surgical patients in local setting.

Literature Review

To summarize and inform the development of the VR based intervention for managing preoperative anxiety, the Preferred Reporting Items for Systematic reviews and Meta-analysis (PRISMA) guidelines was adopted for the literature review (Moher et al., 2009).

Search Strategies

An exhaustive search was conducted on electronic databases, including Allied and Complementary Medicine (AMED), CUHK Full Text Journals, Cumulative Index to Nursing and Allied Health Literature (CINAHL Complete) via EBSCOhost, Cochrane Library, Joanna Briggs Institute EBP Database, EMBASE, Medline via OvidSP, Pubmed, PsychINFO, Scopus, China Journal Net and WanFang Data Chinese Dissertations Database. The search was further

supplemented with bibliographies, Google search and manual search from the reference lists of relevant or similar studies for extending search areas. No date limit was applied to the search, which including literature available from inception (1806) to date of search (April 2021).

Inclusion and Exclusion Criteria

Studies with patients who undergo surgery under anaesthesia with VR-based intervention that comparing to usual care to reduce preoperative anxiety would be included. The search terms of both text words and medical subject heading (MeSH) term with combination of wildcard and truncation of “anxiety”, “preoperative”, and “VR” in either English, Traditional Chinese and Simplified reporting on the effect of interventional studies with VR-based intervention via head-mounted display (HMD) with either fully immersive 3-dimensional computer-generated environment or 360° videos in surround stereoscopic vision were considered eligible for the review. Due to the small number of studies on this topic, since preoperative anxiety is an universal issue across the age, study in children population had also been included for providing a more macroscopic view in this novel approach to facilitate the development and implementation of the intervention. Exclusion criteria were non-randomized controlled trial (RCT) design, non-preoperative intervention, such as intraoperative and postoperative interventions, simulation interventions, such as interventions with visual and audio stimulation but no interaction between user and computer-generated world, non-clinical setting, such as in simulation laboratory setting, and studies with recruited subject did not include the one who undergo surgery under anaesthesia.

Quality Appraisal

The risk of bias of included study were examined according to the Cochrane Handbook for Systematic Review of Interventions in Review Manager (RevMan) [Computer program]

version 5.4. for Mac OS (The Cochrane Collaboration, 2020), in which the methodological quality of trials of randomization sequence generation, allocation concealment, blinding of participants and personnel, blinding of outcome assessment, incomplete outcome data, selective reporting and other bias were evaluated by either ‘low risk’, ‘high risk’ or ‘unclear’.

Result

The combined search yielded 968 studies. Duplicated records had been excluded at the initial screening, which yielded 593 studies for subsequence screening. Based on the information from title and abstract, 542 studies had been excluded, and 51 studies had been retrieved in full-text for assessing compliance with the inclusion and exclusion criteria. Total of 41 studies had been excluded after full-text review, including non-RCT study (n=1), non-interventional study (n=9), non-operative intervention (n=11), non VR intervention (n=11), commentary (n=2) and non-relevant studies (n=6). At the end, 10 studies were included after the quality appraisal. A summary of the literature search had been summarized in figure 1 and the summary of risk of bias of included studies had been summarized in figure 2.

Figure 1.

PRISMA flowchart of study selection

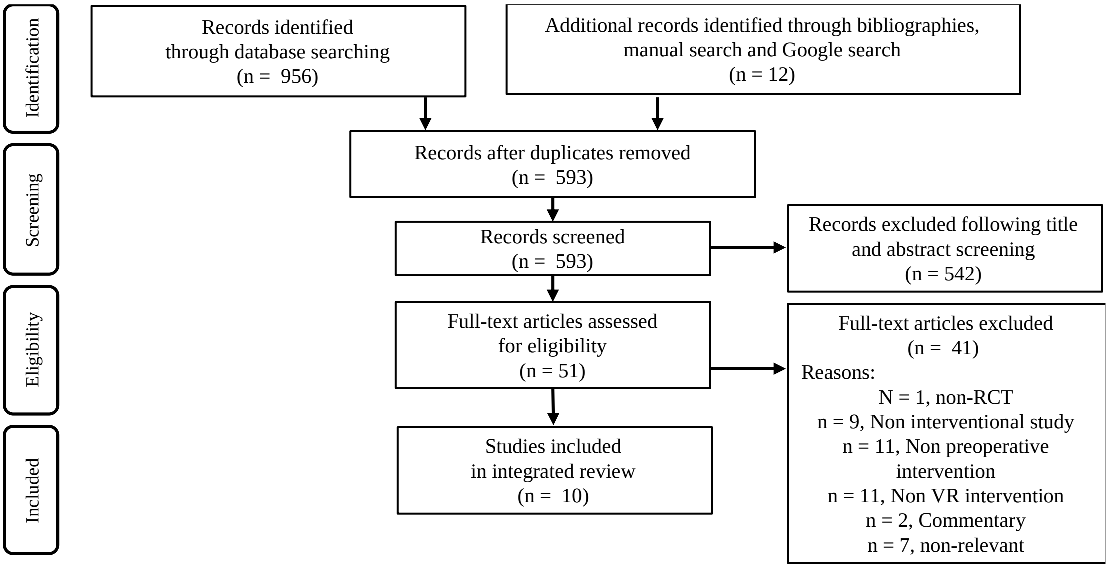

Figure 2.

Summary of Risk of Bias of Included Studies

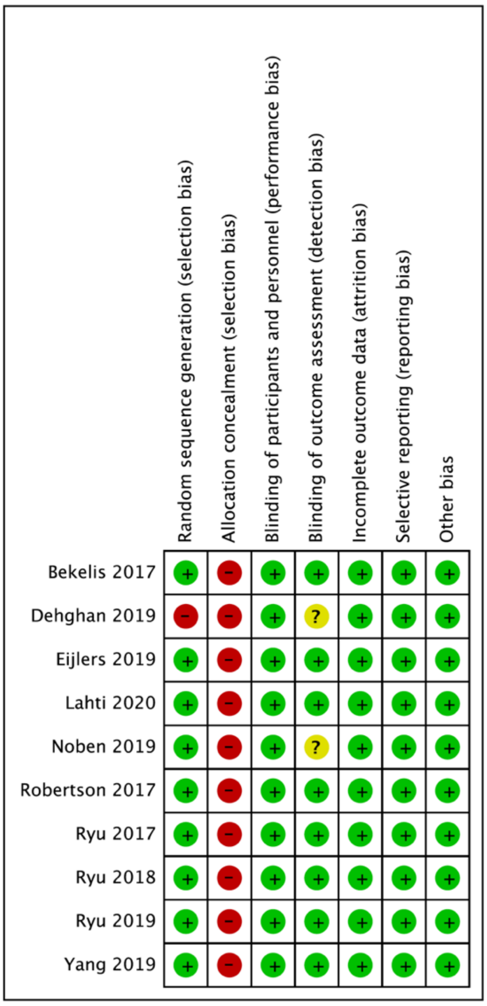

Characteristics of study

Ten included randomized controlled trials were originated from Australia, Finland, Netherland, Iran, South Korea, and United Stated. The study was conducted from 2017 to 2020. Among the 10 studies, a total of 1,036 subject were recruited including 462 male and 547 female. The number of recruited subjects ranged from 40 to 255, with a median of 74.5. A total of five studies of the 449 subjects were children, that is, under 18 years of age, and another six studies of total 587 subjects were adult. The other characteristics including type of surgery, sample size, gender distribution, population and mean age, use of VR devices, approach and treatment intervention, control, intervention dose in duration, measurement tool for preoperative anxiety and key findings was reported in table 1.

Effects of VR-based Intervention on Preoperative Anxiety

The VR-based interventions demonstrated a significant effect to reduce preoperative anxiety by both exposure approach and distraction in adult (Bekelis et al., 2017; Lahti et al., 2020; Yang et al., 2019) and children (Dehghan et al., 2019; Ryu et al., 2017, 2018, 2019), while the interventions were more efficacious in exposure approach and in adult population.

A notably growing number of evidence of VR-based intervention researches across different part of the world unveiled that the preoperative anxiety could be effectively managed using this innovative and newfangled contrivance. Although some studies shown non-significant results in reduction on preoperative anxiety (Eijlers, Dierckx, et al., 2019; Noben et al., 2019; Robertson et al., 2017), the overall intervention effect of VR-based intervention on reducing preoperative anxiety indicated a significant beneficial effect of VR-based intervention over the standard usual care group.

Approach of VR Based Intervention

Two major technological approaches, distraction and exposure, of VR based intervention had been adopted for managing preoperative anxiety.

The assumption for distraction approach was that limited amount of information could be processed at a time by an individual. For an individual immersing in a computer-generated virtual reality environment with visual and audio sensory stimulation, the subjective perception such as pain and anxiety can be reducibly perceived (Eijlers et al., 2019b; Khadra et al., 2018). The distraction would be created by immerging the individual into the virtual reality world, and recruiting the individual in a specific attention task predominantly, in which the attention to anxiety can be limited and restricted (Wahn &König, 2017).

Meanwhile, the exposure approach, as commonly seen for managing claustrophobia and agoraphobia, was to expose the individual into prior experience in virtual environment that similar to real life of potentially phobic virtual environments in a safer, less embarrassing and cost-effective way (Alsina-Jurnet et al., 2011; Dehghan et al., 2019; Klinger et al., 2005; Yeh et al., 2018). Hence, the avoidance and resistance to the anxiety-inducing environment could be reduced (Carvalho et al., 2010; Dehghan et al., 2019).

Regarding to the approach of the VR-based intervention for managing preoperative anxiety, only exposure approach had been adopted for children population, while, however, both exposure and distraction approach had been adopted for adult population. Among the two studies on distraction approach (Lahti et al., 2020; Robertson et al., 2017), both studies distracting the adult subjects by immersive experience with stunning virtual landscape scene, in which it might not be suitable and attractive for children subjects. For the VR-based intervention with exposure approach, contrariwise, provided information on the operation and immersed subjects into a high-fidelity experience of operating theater. Bekelis et al. (2017) adopted the 360° immersive video as the medium for the exposure, in which it provided a vivid

yet cost-effective way in producing the VR intervention. Indeed, study (Karimi et al., 2014) unveiled a significant reduction of anxiety, pain level following implementation of orientated tour to operating theater, while physical tour would not be as simple as conducting in VR environment, due to labour intensive, prior arrangement and contamination concerns, in which the feasibility and practicability of implementation in clinical setting would be inevitably jeopardized.

Devices use in the VR-based Interventions

With the evolution of virtual reality technology to smaller, more compact but yet powerful system, wearable and lightweight virtual reality system becomes useable, accessible and affordable in healthcare settings (Greengard, 2019; Parsons et al., 2009). Although the high-end professional, and yet exorbitant in cost, VR headset could provide with a superior graphic processing power with generation of higher definition image, as revealed from the included studies, a consumer level all-in-one HMD with build-in display, such as Oculus Go, Quest and Quest 2 (Facebook, 2020), or even smartphone equipped HMD, such as Samsung Gear VR (Samsung, 2019) and Google Cardboard market (Google, 2020), could already able to render high-fidelity virtual reality image and would be sufficiently applied in clinical setting with a promising result. These devices were all equipped with head-motion tracking system that provide fully immersive experience, while most, if not all, devices could be equipped with separated handheld controller enhance the capability to interact with the VR world.

Although six studies had adopted computer-connected VR head-mounted display (HMD) with built-in display units, which included HTC Vive HMD (Eijlers, Dierckx, et al., 2019; Yang et al., 2019), Oculus VR device or Oculus Riff (Bekelis et al., 2017; Ryu et al., 2018), and unmentioned brand VR eyeglasses (Dehghan et al., 2019), these devices are wired and might be a challenge in deployment in clinical setting. Furthermore, other seven studies

adopted VR HMD required additional smartphone devices as display units, which included Samsung Gear VR (Lahti et al., 2020; Robertson et al., 2017; Ryu et al., 2017, 2019), and unmentioned VR eyeglasses (Dehghan et al., 2019; Noben et al., 2019), in which the quality might not be desirable.

With the consideration of cost of devices, software development, portability, and devices deployment, standalone HMD would be a desirable option for implementation in most of the general clinical setting by nursing team and other healthcare profession, and for subject to effortlessly operating through simple head and body movement.

Summary

To summarize the intervention design of the included studies, the exposure approach should be adopted for adult subject, as a more efficacious reduction on preoperative anxiety were resulted. A VR-based intervention could be designed for the future study in exposure approach, by immersing the subjection into a virtual environment using a immersive 360° video operating theater tour and providing information on the operation and anesthesia experience. With reference to the aforementioned duration of the intervention, the VR-based intervention could be adopted in a duration about 6 minutes.

Unfortunately, there was no gold standard in measuring preoperative anxiety and therefore different measuring tools had been adopted in the included studies.

For the measurement tool of preoperative anxiety for adult, the APAIS was a validated tool covered both monitor and blunting aspects with four items assessing fear of anaesthesia and fear of surgical procedure and two items assessing the need for information (Moerman et al., 1996). The HADS questionnaire was also a validated tool with 14 questions, in which the sum of each items, that containing four possible answers with a score between 0 to 3, scoring the subject to be normal (0 to 7), borderline abnormal (8 to 10), and abnormal (11 to 21) (Snaith,

2003). The MDAS was a validated tool having 5 questions specifically describing the dental anxiety which each of them with five reply alternatives from not anxious to extremely anxious (Humphris et al., 1995). The visual analogue scale in the aforementioned studies comprised a horizontal line to rate the preoperative anxiety by stating the position between least anxious to most anxious, in which no definitively validity had been established.

This literature review imposed limitations of which there were only limited studies available for adult population. Also, despite the vast variety of sample size, measuring tool, VR application and approach, the meta-analysis could not be conducted due to great heterogeneity. Considering the nature of the intervention, most of the study could only adopt a single blinded design.

Theoretical Framework

The study adopts Lazarus and Folkman (1987) Transactional Theory of Stress and Coping as the theoretical framework. According to the theory, stress was neither situated in the individual nor the environment per se, but the result of the mutual influence and relationship of the interaction between an individual and the external environment. An individual constantly evaluate and appraise the environment and attempt to cope with the challenges ahead.

The primary appraisal is focused on cognitive, that concerns the motivational relevance and implications of the well-being of an individual dealing with the environment, which harm experienced, threat anticipated and challenge potentially mastery or gain resulting the stress. Therefore, an individual conducts cognitive appraisal to the stressful event for the impact to self.

If the stressful event is regarded as an threat or challenge, an individual conduct the secondary process to evaluate the possible outcomes of the challenges and actions to deal with the event (Cheng &Cheung, 2005). The second appraisal evaluates the external and internal

resources by altering cognitive and behavioural efforts to cope with the challenge ahead. In case of insufficient of the available resource, the coping involve two distinctions, in which problem focused coping focusing on solve the demands of the stress by change or modify the stress inducing situation such as gathering more information; and emotional response to the stressful condition would be regulated for an individual through emotion-focused coping strategies, for instances, minimization, avoidance, distancing selective attention and finding positive value in negative events, etc (Chirico et al., 2020). Hence, distress should not be experience when an individual perceives having the ability to cope with the threatening situation, while distress could be arise when an individual perceives the demands are exceed the available resources which threatening to the well-being to the specific situations, that a functional change for an individual might be needed to return the balance.

Figure 3.

Flowchart of Transactional Theory of Stress and Coping

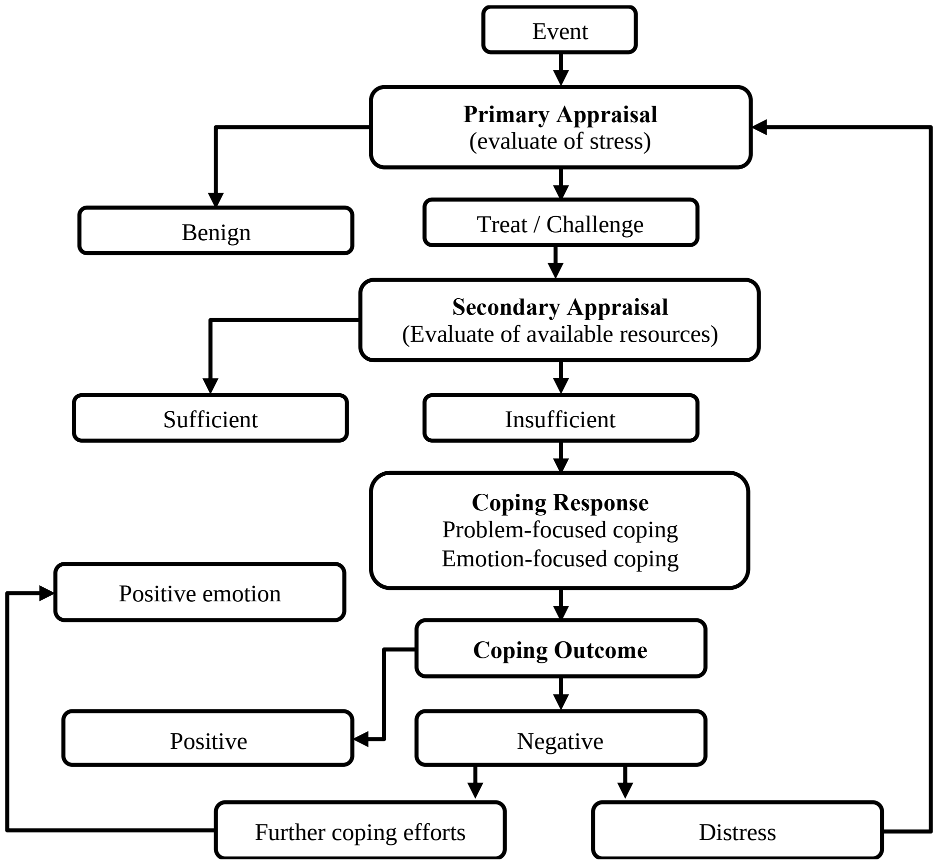

The dynamic of the coping of stress was illustrated in the flowchart in figure 1 (Turner-Cobb &Hawken, 2019). In summary, the imbalance between demands and resources and perceived capability of dealing with the external environment causes the stress to an individual, when stress responses are controllable when resources and abilities to cope is mediated. By providing exposure and information regarding to the perioperative environment through the VR based intervention, negative impact on the preoperative anxiety would be improved.

Service Gap

To address the preoperative anxiety, existing interventions imposed limitations in the current situation, in which providing information through patient education in text-based or video-based format could not satisfy the needs of the patients in the modern era, while preoperative orientation tour would impose an inevitable challenge in view of feasibility and practicability of implementation in clinical setting. Evidence demonstrated VR based intervention could effectively manage preoperative anxiety, while it is still uncertain that if VR based intervention with preoperative education could be benefit to adult population. To the best knowledge, there was no local study conducted in evaluating VR based intervention, from development to implementation, and effect in reducing preoperative anxiety. The study could identify the effect and feasibility on reducing preoperative anxiety via VR based intervention in local setting.

Study Aim and Objectives

The study aims to examine the effectiveness of VR based intervention with preoperative education to reduce preoperative anxiety among adult patients undergoing surgery.

The primary objective of the study was to investigate the effect of the VR based intervention with preoperative education on preoperative anxiety. The other objectives was to

investigate the effect of the intervention on other outcomes, including postoperative pain, improvement in preparedness for the surgery, stress, anaesthesia requirement, and the feasibility and potential to implement VR based intervention in clinical settings.

Study Design

This study will adopt prospective, single-blind randomized controlled trial (RCT) for evaluating intervention effectiveness that bias could be reduced by means of randomization in subject allocation (Hariton &Locascio, 2018). Subjects will be equally and randomly assigned to either the interventional group (VR based intervention) or the control group (usual standard care) with remaining the same chances, to assure that the characteristics of the participants are as likely to be similar as possible across groups at the start of the comparison.

The subjects and the outcome assessor will be blinded to the group allocation, while the interventionist in this trial cannot be blinded to the allocation of treatment groups due to the nature of the intervention. Subject will be recruited without any knowledge of participant allocation and therefore considered safe from bias.

Subjects attend the Pre-anaesthesia Assessment Clinic will be invited and to be recruited. The inclusion criteria for this study are set as follows:

Patients who

i. is an adult who at least 18 years old;
ii. scheduled with an elective surgery undergoing general anaesthesia in the coming 2 to 4 week;
iii. can speak Cantonese;
iv. attend the Pre-anaesthesia Assessment Clinic for the scheduled elective surgery; and
v. is American Society of Anaesthesiologists Grade I or II.

The exclusion criteria for this study are set as follows:

Patients who

i. is undergoing emergency procedure without prior preoperative assessment;
ii. has previous experience of surgical operation or expose to preoperative experience;
iii. is cognitive impaired or unable to consent;
iv. has history of vestibular dysfunction or motion sickness;
v. has history of any psychological disorder;
vi. has history of alcohol or substance abuse; or
vii. has Parkinson's disease, multiple sclerosis or muscular dystrophy.

After consenting to participate into the study, the baseline data will be collected to access the eligibility. Block randomization will be adopted in the study, while subjects will be randomized into predetermined groups that result in equal sample sizes block. A block of four in 1:1 ratio, in six possible sequences (i.e. IICC, CCII, ICIC, CIIC, ICCI, CICI) will be randomly selected for subject allocation to the intervention group (I) and the control group (C). Serial numbered opaque sealed envelopes containing the group identifier (I or C) will be prepared by the research team using computer generated random codes. Block randomization has been adopted to ensure a balance in sample sizes between intervention group and control group and across groups over time (Suresh, 2011).

Sampling Method and Sample Size Planning

Consecutive sampling will be adopted in this study. Adult subjects, age at least 18 years old, who attend the pre-anaesthetic assessment clinic for scheduled elective surgery undergoing general anaesthesia and consented to the study, are eligible to the VR based intervention. The

elective surgery refers to planned surgical procedure that happening at least 48 hours after initial assessment. Every subject meeting inclusion criteria will be invited to the study until required sample size is achieved. Consecutive sampling is better in controlling selection and sampling bias and minimizing volunteerism (Hulley et al., 2007).

With reference to the meta-analysis of 10 RCTs on the effect of VR on preoperative anxiety (Koo et al., 2020), a sample size of at least 45 per group (i.e. total 90) will be sufficient to detect an effect size of 0.64 between intervention and control groups, with a statistical power of 80% and an alpha of 5%, allowing for 10% of drop-out rate.

Intervention

Subject in the intervention group will receive the VR based intervention.

VR based Intervention and Equipment

Subject will watch a 5 to 6 min 360° immersive VR video through an all-in-one VR console (Oculus Quest 2) in a separated room in Pre-anaesthesia Assessment Clinic during the visit. The Oculus Quest 2 (Facebook, 2020) is an all-in-one standalone Head-Mount-Display VR Console, in which

- operated without attachment of any computers or device;
- screen resolution of 1832 x 1920;
- refresh rate at 90 Hz;
- integrated speakers and microphone;
- 2 to 3 hours battery life; and
- adjustable inter-pupillary distance with three settings for 58, 63 and 68mm.

Content of the VR Based Video

With reference to the previous studies of providing intervention with VR tour of operating theatre (Bekelis et al., 2017; Dehghan et al., 2019; Eijlers, Dierckx, et al., 2019; Noben et al., 2019; Ryu et al., 2017, 2019), a tailor-made 360° VR video would be produced for the subject immersing into a virtual environment using a 360° video operating theater tour and providing information on the operation and anesthesia experience of a scheduled surgery. There would be four sections of the story, and each sections will be narrated with actor/actress to introduce and explain the peri-operative process. The narrator will emphasize in the VR video that all subjects will have similar experience and undergo similar process.

The story will be begun in ward area, aiming to let the subject experiencing the preparation prior to the operation, that a ward nurse will perform the pre-operative checking procedures on the subject, assist the subject to change the surgical gown, and ask the subject to stay on patient transport trolley, while the patient will be transferred from ward to reception area of the operating theatre with escort nurse through hospital corridors and doors.

The second sections will be begun in the reception area of operating theatre, aiming to let the subject experiencing the workflow on how the admission and validation perform, that the nurse from operating theatre will perform peri-operative checking procedures on the subjects, such as confirming identification of the subject, asking name of the operating procedure, verifying the consent signed for the operation, and afterward the subject will be transferred to induction area of the operating theatre.

The third sections will be begun in the induction area of the operating theatre and transferring into inside the theatre room, aiming to let the subject experiencing the checking procedure, exposing the setting, environment, equipment and staff inside the operating theatre, that the surgeon, anaesthetist and operating theatre nurse will perform checking procedure and be explained the staff and equipment. The anaesthetist will then set an intravenous access in the subject dorsum area, after monitoring devices, including non-invasive blood pressure

measuring cuff, pulse oximeter, and cardiac monitor leads has been attached. The subject will be put on a face mask with flowing sound of anaesthetic gas and the screen will be turning dim after the anaesthetist asking the subject to close the eyes.

The last sections will be begun with a black screen with lots of sound inside operating theatre room, aiming to let the subject expose the experience from theatre room to recovery area, that the subject will be asked by the anaesthetist to eyes opening and the screen will turn bright gradually. The subject will then be transferred to recovery area, be assessed the vital sign of the patients by nurse in recovery area, and be handover to ward nurse and be transferred back to ward through hospital door and corridors.

While the average duration of the similar studies of adopting exposure approach (Bekelis et al., 2017; Dehghan et al., 2019; Eijlers, Dierckx, et al., 2019; Noben et al., 2019; Ryu et al., 2017, 2018, 2019; Yang et al., 2019) was 6.11 minutes, ranging from 4 to 15 minutes, the VR-based intervention would be adopted in a duration about 6 minutes and not more than 15 minutes.

Script of the video were written with vetting by expert panel including a Consultant of Anaesthesiology, the Department Operation Manager of Operating Theatre, and a Nurse Specialist of Perioperative Nursing. Video was in a 360° format in around 5 to 6 min, which will be produced by professional service, utilizing Nurses and Doctors worked in OT as actors describing the experience of a patient undergoing uncomplicated surgery with general anaesthesia from preoperatively to postoperatively. Subject will have immersive, vivid yet realistic experience of the operating theatre with a narrative description provided by the actor explaining steps in the operating theatre.

Control

Routine text-based or video descriptions of preoperative information will be provided to subject in the control group. Concerns and questions will also be answered by the healthcare professional in the clinic.

Data collection methods and procedures

Upon scheduled an elective surgery, the patient will be arranged an assessment in the Pre-anaesthesia Assessment Clinic. After screening of the eligibility according to the attendance list provided by the clinic, subject will be invited to participating in the study. And after consenting to the study, a research team member will take an envelope contain grouping identifier (I or C) to allocate recruited subjects to either intervention or control group. Intervention group will receive VR based intervention and control group received usual standard care.

Baseline data will be collected at the end of the clinic session (T0), and afterward, the intervention group will be asked to sit in the chair and stay in a separated cubicle within clinic and provided with the HMD with disposable cover and received the VR intervention and the control group will be asked to sit in the chair and stay in the activity room with standard preoperative education materials and instruct to read. After about 10 min, subject will be asked if there would be any further question and will be re-assessed of post intervention data (T1). Subjects will be then be discharged and will be admitted on the day of OT, usually within 1 to 2 weeks. Second set of data would be assessed on the day of OT (T2). After OT, subjects in both intervention group and control group will be assessed of the third set of post-intervention data (T3) at 24 to 48 hour after surgery.

Subjects' socio-demographic and clinical data will be collected by custom-designed questionnaire, subjects' clinical charts and computerized hospital records at baseline. The data

include age, gender, marital status, living arrangement, educational level current medication regimen, past medical history. Preoperative anxiety level and the pain level, preparedness, stress, anaesthesia requirement, postoperative analgesic use and length of stay will be collected accordingly at different time points. The arrangement is summarized in figure 4.

Figure 4.

SPIRIT flow diagram of the Study

	**STUDY PERIOD**				
	**Enrolment and Allocation**	**Post-allocation**			**Close-out**
**TIMEPOINT****	***-t1***	***T0*** *Pre-intervention*	***T1*** *Post-intervention*	***T2*** *Preoperative*	***T3*** *Post-Operative*
**ENROLMENT:**					
**Eligibility screen**	X				
**Informed consent**	X				
**Allocation**	X				
**INTERVENTIONS:**					
[VR Based Intervention]		X			
[Usual Standard Care]		X			
**ASSESSMENTS:**					
Baseline	X				
APAIS		X	X	X	
Simulation Sickness		X	X		
Pain			X	X	X
Stress		X	X	X	
Preparedness		X	X	X	
Anaesthesia Requirement					X
Post-operative Analgesic Use					X
Length of Stay					X

Outcome Assessments and Instrumentation

Primary Outcome

Preoperative Anxiety

The preoperative anxiety will be measured by Amsterdam Preoperative Anxiety and Information Scale (APAIS), which is developed by (Moerman et al., 1996). It is a self-report questionnaire to quickly assess preoperative anxiety. The six items were divided into two subscales (Anxiety Scale and Need-for-information Scale). The instrument had a very good internal validity of RMSEA 0.069 and CFI 1.0. The Cronbach's alpha for the anxiety scale was 0.86 and for the need-for-information scale was 0.68. A literature review (Sarah &Lynn, 2019) of 7 RCTs concluded that APAIS is a valid instrument for assessing preoperative anxiety, and highly correlated with State-Trait Anxiety Inventory-State (STAI-S) (>0.60) and more specific to preoperative assessment. The Chinese version of APAIS validated with Cronbach's alpha 0.862 and 0.830 for anxiety and need-for-information scale; RMSEA 0.073 (Wu et al., 2020). The data would be collected at T0, T1 and T2.

Secondary Outcomes

Simulation Sickness

The simulation sickness will be assessed by the Simulation Sickness Questionnaire, which developed by Kennedy et al. (1993). It is a self-report questionnaire contains 16 symptoms related to simulator sickness with 4-point rating scale into 3 clusters, in which

- Nausea: Cronbach's alpha 0.84;
- Oculomotor: Cronbach's alpha 0.91;
- Discomfort: Cronbach's alpha 0.88.

The instrument was the gold standard for assessing sickness after simulation or VR environment and adopted by over 3000 VR research (Bimberg et al., 2020). A higher score represents a greater level of simulator sickness. The Chinese translated version, adopted by VR research in Chinese population (Chen et al., 2011; Ng, 2002), with expert panel review for content validation, would be used and be used to collect data at T0 and T1.

Pain, stress and preparedness

Self-report Visual Analog Scale with continuous scale of a horizontal line in 100 mm in length, anchored by 2 verbal descriptor of symptom extreme would be used for assessing the pain level, stress level and preparedness. It is quick to administer and widely adopted for assessing aforementioned which is more sensitive to identify fine changes then solely numerical scales and four point scales. The data of pain would be collected at T1, T2 and T3, while the stress and preparedness level would be collected at T0, T1 and T2. The anaesthesia requirement, postoperative analgesic use and length of stay will be collected through review of patient document at T3.

Data analysis plan

Intention-to-treat analysis would be adopted in this study. Every subject who is randomized in this study would be included in the analysis. Non-compliance, protocol deviations, withdrawal or anything that happens after randomization will be ignored. The advantage of adopting intention-to-treat analysis is it could maintain prognostic balance generated from the original random treatment allocation and could also generate an unbiased estimation of treatment effect (Gupta, 2011).

The data will be entered in the statistical computer software, IBM SPSS Statistics for Macintosh (Version 27.0). Subject characteristics and baseline outcome measures will be

summarized by frequency, percentage, mean, standard deviation and range. All tests of significance were 2-sided. For comparing subject characteristics between intervention and controls groups, chi-square test and independent samples t test will be adopted, independent sample t-test and ANOVA will be adopted for comparing the preoperative anxiety level, pain level, stress level, preparedness, postoperative analgesic use, length of stay, and for comparing anaesthesia requirement, Wilcoxon signed rank test will be used for simulation sickness before and after the intervention, and generalized estimating equations model will be used in subgroup analysis.

Pilot Study

A pilot study will be conducted in order to examine the feasibility of the delivery of intervention and identify potential problem areas and deficiencies in the data collection. It also aim to refine the elements of the interventions (Hassan et al., 2006).

A pilot study will be conducted as soon as ethical approval is obtained. Before the main trial, a feasibility and pilot study will be conducted to assess the feasibility, in term of recruitment rate, eligibility, consent rate, withdrawal rate, attrition rate and the preliminary effect of the VR based intervention on preoperative anxiety. At least 9% of sample size of main trial should be recruited for the pilot randomized study (Cocks &Torgerson, 2013). Therefore, as 90 subjects was planned to be recruited in the main trial, about 10 subjects for the pilot study should be sufficient

Timetable of work

The timetable of the study were listed in the table below:

Table 1.

Timetable of work

Key Tasks	Feb21	Mar	Apr	May	Jun	Jul	Aug	Sep	Oct	Nov	Dec	Jan22	Feb
Literature review	X	X											
Develop research proposal		X	X	X	X								
VR Video Production					X	X							
Ethical Approval					X	X	X	X					
Feasibility and Pilot Study								X	X				
Data Collection									X	X	X	X	
Data Analysis												X	
Write Up Dissertation												X	X

Ethical issues

The study will be commenced after approved to conduct the study granted by Hospital Authority New Territory West Cluster Research Ethic Committee and the Joint CUHK-NTEC Ethics Committee. The study will comply Code of ethics and guidelines by the Declaration of Helsinki and Good clinical practice (GCP) from International Conference of Harmonisation (ICH). Written informed consent will be obtained prior to data collection from each subject. For assuring the participants having adequate information and time to make their decision, detail explanation on the purpose of the research, involvement in the study, expected risks and benefits; and ways of handling their information, will be stipulated in the informed consent. The name and contact telephone number of the researcher will be provided to each participant for follow up and subsequent inquiry about the study. The principal investigator will ensure and reinforce the voluntary basis of participation and highlight the right of withdrawal, for whatever reason, at any time without recrimination or prejudice.

The confidentiality and anonymity of the participants' information will be assured and all data would be handled in line with Hospital's policy in handling, storage, and destruction of subject's medical records. Collected data could only be accessed by the principal investigator and his research supervisors in need to know basis. No individual subject will identifiable for information published or presented in relation to this study. Unique code will be generated to each participants for identification, and therefore subject will not be identified by name on any of the data sheets. The researcher will be responsible for collecting and analysing the information. The data and questionnaires will be destroyed at around five year upon the completion of the study.

Significance of the study for nursing practices

High prevalence of preoperative anxiety with limited approach of intervention lead to adverse outcomes & burden to patient, organization and healthcare system, option while VR technology provides an opportunity to improve the situation. Existing evidences showed effective to manage preoperative anxiety by VR based intervention for both adult and children. To the best knowledge, there was no local study conducted in evaluating VR based intervention, from development to implementation, and effect in reducing preoperative anxiety. The study could identify the effect and feasibility on reducing preoperative anxiety via VR based intervention in local setting.

References

Alanazi, A. A. (2014). Reducing anxiety in preoperative patients: A systematic review. In *British Journal of Nursing* (Vol. 23, Issue 7, pp. 387–393). https://doi.org/10.12968/bjon.2014.23.7.387

Almalki, M. S., Hakami, O. A. O., &Al-Amri, A. M. (2017). Assessment of Preoperative Anxiety among Patients Undergoing Elective Surgery. *The Egyptian Journal of Hospital Medicine*, *69*(4), 2329–2333. https://doi.org/10.12816/0041537

Alsina-Jurnet, I., Gutiérrez-Maldonado, J., &Rangel-Gómez, M. V. (2011). The role of presence in the level of anxiety experienced in clinical virtual environments. *Computers in Human Behavior*, *27*(1), 504–512. https://doi.org/10.1016/j.chb.2010.09.018

Ayral, X., Gicquere, C., Duhalde, A., Boucheny, D., &Dougados, M. (2002). Effects of video information on preoperative anxiety level and tolerability of joint lavage in knee osteoarthritis. *Arthritis & Rheumatism*, *47*(4), 380–382. https://doi.org/10.1002/art.10559

Battah, H., Alzoraigi, U., &Shubbak, F. (2021). Effectiveness of preoperative tour to a simulated anaesthesia induction at operating theatre in reducing preoperative anxiety in children and their parents: A pragmatic, single-blinded, randomised controlled trial/ King Fahad Medical City. *BMJ Simulation and Technology Enhanced Learning*, bmjstel-2020-000707. https://doi.org/10.1136/bmjstel-2020-000707

Bauer, K. P., Dom, P. M., Ramirez, A. M., &O'Flaherty, J. E. (2004). Preoperative intravenous midazolam: Benefits beyond anxiolysis. *Journal of Clinical Anesthesia*, *16*(3), 177–183. https://doi.org/10.1016/j.jclinane.2003.07.003

Bedaso, A., &Ayalew, M. (2019). Preoperative anxiety among adult patients undergoing elective surgery: A prospective survey at a general hospital in Ethiopia. In *Patient Safety*

in Surgery (Vol. 13, Issue 1). BioMed Central Ltd. https://doi.org/10.1186/s13037-019-0198-0

Bekelis, K., Calnan, D., Simmons, N., Mackenzie, T. A., &Kakoulides, G. (2017). Effect of an immersive preoperative virtual reality experience on patient reported outcomes: A randomized controlled trial. *Annals of Surgery*, *265*(6), 1068–1073. https://doi.org/10.1097/SLA.0000000000002094

Bellew, M., Atkinson, K. R., Dixon, G., &Yates, A. (2002). The introduction of a paediatric anaesthesia information leaflet: An audit of its impact on parental anxiety and satisfaction. *Paediatric Anaesthesia*, *12*(2), 124–130. https://doi.org/10.1046/j.1460-9592.2002.00788.x

Bimberg, P., Weissker, T., &Kulik, A. (2020). On the Usage of the Simulator Sickness Questionnaire for Virtual Reality Research. *Proceedings - 2020 IEEE Conference on Virtual Reality and 3D User Interfaces, VRW 2020*, 464–467. https://doi.org/10.1109/VRW50115.2020.00098

Bradt, J., Dileo, C., &Shim, M. (2013). Music interventions for preoperative anxiety. *Cochrane Database of Systematic Reviews*, *CD006908*(6). https://doi.org/10.1002/14651858.CD006908.pub2

Calvin, R. L., &Lane, P. L. (1999). Perioperative uncertainty and state anxiety of orthopaedic surgical patients. *Orthopaedic Nursing / National Association of Orthopaedic Nurses*, *18*(6), 61–66. https://doi.org/10.1097/00006416-199911000-00011

Carvalho, M. R.De, Freire, R. C., &Nardi, A. E. (2010). Virtual reality as a mechanism for exposure therapy. In *World Journal of Biological Psychiatry* (Vol. 11, Issue 2 PART 2, pp. 220–230). https://doi.org/10.3109/15622970802575985

Caumo, W., Schmidt, A. P., Schneider, C. N., Bergmann, J., Iwamoto, C. W., Adamatti, L. C., Bandeira, D., &Ferreira, M. B. C. (2001). Risk factors for postoperative anxiety in adults. *Anaesthesia*, *56*(8), 720–728. https://doi.org/10.1046/j.1365-2044.2001.01842.x

Celik, F., &Edipoglu, I. S. (2018). Evaluation of preoperative anxiety and fear of anesthesia using APAIS score. *European Journal of Medical Research*, *23*(1), 41. https://doi.org/10.1186/s40001-018-0339-4

Chen, W., Chen, J. Z., &So, R. H. Y. (2011). Visually induced motion sickness: Effects of translational visual motion along different axes. *Contemporary Ergonomics and Human Factors 2011*, 281–287. https://doi.org/10.1201/b11337-40

Cheng, C., &Cheung, M. W. L. (2005). Cognitive processes underlying coping flexibility: Differentiation and integration. *Journal of Personality*, *73*(4), 859–886. https://doi.org/10.1111/j.1467-6494.2005.00331.x

Chirico, A., Maiorano, P., Indovina, P., Milanese, C., Giacomo Giordano, G., Alivernini, F., Iodice, G., Gallo, L., DePietro, G., Lucidi, F., Botti, G., DeLaurentiis, M., &Giordano, A. (2020). Virtual reality and music therapy as distraction interventions to alleviate anxiety and improve mood states in breast cancer patients during chemotherapy. *J Cell Physiol*, *235*, 5353–5362. https://doi.org/10.1002/jcp.29422

Cocks, K., &Torgerson, D. J. (2013). Sample size calculations for pilot randomized trials: A confidence interval approach. *Journal of Clinical Epidemiology*, *66*(2), 197–201. https://doi.org/10.1016/j.jclinepi.2012.09.002

Croke, L. (2020). Nonpharmacologic strategies to help reduce preoperative patient anxiety. *AORN Journal*, *111*(2), P8–P10. https://doi.org/10.1002/aorn.12970

Dehghan, F., Jalali, R., &Bashiri, H. (2019). The effect of virtual reality technology on preoperative anxiety in children: a Solomon four-group randomized clinical trial. *Perioperative Medicine*, *8*(1), 1–7. https://doi.org/10.1186/s13741-019-0116-0

Eijlers, R., Dierckx, B., Staals, L. M., Berghmans, J. M., Van DerSchroeff, M. P., Strabbing, E. M., Wijnen, R. M. H. H., Hillegers, M. H. J. J., Legerstee, J. S., &Utens, E. M. W. J. W. J. (2019). Virtual reality exposure before elective day care surgery to reduce anxiety and pain in children: A randomised controlled trial. *European Journal of Anaesthesiology*, *36*(10), 728–737. https://doi.org/10.1097/EJA.0000000000001059

Eijlers, R., Utens, E. M. W. J. W. J., Staals, L. M., DeNijs, P. F. A. A., Berghmans, J. M., Wijnen, R. M. H. H., Hillegers, M. H. J. J., Dierckx, B., &Legerstee, J. S. (2019). Systematic Review and Meta-analysis of Virtual Reality in Pediatrics: Effects on Pain and Anxiety. *Anesthesia and Analgesia*, *129*(5), 1344–1353. https://doi.org/10.1213/ane.0000000000004165

Facebook. (2020). *Oculus | VR Headsets & Equipment*. https://www.oculus.com/

Filatov, S. M., Baer, G. A., Rorarius, M. G. F., &Oikkonen, M. (2000). Efficacy and safety of premedication with oral ketamine for day-case adenoidectomy compared with rectal diazepam/diclofenac and EMLA® . *Acta Anaesthesiologica Scandinavica*, *44*(1), 118–124. https://doi.org/10.1034/j.1399-6576.2000.440121.x

Foronda, C. L., Alfes, C. M., Dev, P., Kleinheksel, A. J., Nelson, D. A., O'Donnell, J. M., &Samosky, J. T. (2017). Virtually Nursing: Emerging Technologies in Nursing Education. *Nurse Educator*, *42*(1), 14–17. https://doi.org/10.1097/NNE.0000000000000295

Freina, L., &Ott, M. (2015). A literature review on immersive virtual reality in education: State of the art and perspectives. *Proceedings of ELearning and Software for Education (ELSE)(Bucharest, Romania, April 23--24, 2015)*, 8. https://doi.org/10.12753/2066-026X-15-020

Google. (2020). *Get Cardboard – Google VR*. https://arvr.google.com/cardboard/get-cardboard/

Greengard, S. (2019). *Virtual Reality*. MIT Press. https://books.google.com.hk/books?id=l53bxQEACAAJ

Guo, P. (2015). Preoperative education interventions to reduce anxiety and improve recovery among cardiac surgery patients: A review of randomised controlled trials. In *Journal of Clinical Nursing* (Vol. 24, Issues 1–2, pp. 34–46). Blackwell Publishing Ltd. https://doi.org/10.1111/jocn.12618

Gupta, S. (2011). Intention-to-treat concept: A review. *Perspectives in Clinical Research*, *2*(3), 109. https://doi.org/10.4103/2229-3485.83221

Hariton, E., &Locascio, J. J. (2018). Randomised controlled trials – the gold standard for effectiveness research: Study design: randomised controlled trials. In *BJOG: An International Journal of Obstetrics and Gynaecology* (Vol. 125, Issue 13, p. 1716). Blackwell Publishing Ltd. https://doi.org/10.1111/1471-0528.15199

Hassan, Z. A., Schattner, P., &Mazza, D. (2006). Doing A Pilot Study: Why Is It Essential? *Malaysian Family Physician : The Official Journal of the Academy of Family Physicians of Malaysia*, *1*(2–3), 70–73. http://www.ncbi.nlm.nih.gov/pubmed/27570591

Hoirisch-Clapauch, S. (2018). Anxiety-Related Bleeding and Thrombosis. *Seminars in Thrombosis and Hemostasis*, *44*(7), 656–661. https://doi.org/10.1055/s-0038-1639501

Hounsome, J., Lee, A., Greenhalgh, J., Lewis, S. R., Schofield-Robinson, O. J., Coldwell, C. H., &Smith, A. F. (2017). A systematic review of information format and timing before scheduled adult surgery for peri-operative anxiety. In *Anaesthesia* (Vol. 72, Issue 10, pp. 1265–1272). Blackwell Publishing Ltd. https://doi.org/10.1111/anae.14018

Hulley, S. B., Newman, T. B., &Cummings, S. R. (2007). Choosing the study subjects: specification, sampling, and recruitment. *Designing Clinical Research*, *3*, 27–36.

Humphris, G. M., Morrison, T., &Lindsay, S. J. (1995). The Modified Dental Anxiety Scale: validation and United Kingdom norms. *Community Dental Health*.

Kain, Z. N., &Maranets, I. (2000). Preoperative Anxiety and Intraoperative Anesthetic Requirements. *Anesthesia & Analgesia*, *91*(1), 250. https://doi.org/10.1213/00000539-200007000-00063

Kain, Z. N., Mayes, L. C., Cicchetti, D.V., Bagnall, A. L., Finley, J. D., &Hofstadter, M. B. (1997). The Yale Preoperative Anxiety Scale: How does it compare with a "gold standard"? *Anesthesia and Analgesia*, *85*(4), 783–788. https://doi.org/10.1097/00000539-199710000-00012

Kain, Z. N., Mayes, L. C., Cicchetti, D.V., Caramico, L. A., Spieker, M., Nygren, M. M., &Rimar, S. (1995). Measurement Tool for Preoperative Anxiety in Young Children: The Yale Preoperative Anxiety Scale. *Child Neuropsychology*. https://doi.org/10.1080/09297049508400225

Karimi, R., Fadaiy, Z., Nikbakht Nasrabadi, A., Godarzi, Z., &Mehran, A. (2014). Effectiveness of orientation tour on children's anxiety before elective surgeries. *Japan Journal of Nursing Science*, *11*(1), 10–15. https://doi.org/10.1111/j.1742-7924.2012.00223.x

Kennedy, R. S., Lane, N. E., Berbaum, K. S., &Lilienthal, M. G. (1993). Simulator Sickness Questionnaire: An Enhanced Method for Quantifying Simulator Sickness. *The International Journal of Aviation Psychology*, *3*(3), 203–220. https://doi.org/10.1207/s15327108ijap0303_3

Khadra, C., Ballard, A., Déry, J., Paquin, D., Fortin, J. S., Perreault, I., Labbe, D. R., Hoffman, H. G., Bouchard, S., &LeMay, S. (2018). Projector-based virtual reality dome environment for procedural pain and anxiety in young children with burn injuries: A pilot study. *Journal of Pain Research*, *11*, 343–353. https://doi.org/10.2147/JPR.S151084

Klinger, E., Bouchard, S., Légeron, P., Roy, S., Lauer, F., Chemin, I., &Nugues, P. (2005). Virtual reality therapy versus cognitive behavior therapy for social phobia: A preliminary controlled study. *Cyberpsychology and Behavior*, *8*(1), 76–88. https://doi.org/10.1089/cpb.2005.8.76

Koo, C.-H., Park, J.-W., Ryu, J.-H., &Han, S.-H. (2020). The Effect of Virtual Reality on Preoperative Anxiety: A Meta-Analysis of Randomized Controlled Trials. *Journal of Clinical Medicine*, *9*(10), 3151. https://doi.org/10.3390/jcm9103151

Kumar Kar, S., &Ganguly, T. (2015). Preoperative Anxiety in Pediatric Population: Anesthesiologist"s Nightmare. *Translational Biomedicine*, *6*(4), 1–9. https://doi.org/10.21767/2172-0479.100030

Lahti, S., Suominen, A., Freeman, R., Lähteenoja, T., &Humphris, G. (2020). Virtual Reality Relaxation to Decrease Dental Anxiety: Immediate Effect Randomized Clinical Trial. *JDR Clinical and Translational Research*, 2380084420901679. https://doi.org/10.1177/2380084420901679

Lazarus, R. S., &Folkman, S. (1987). Transactional theory and research on emotions and coping. *European Journal of Personality*, *1*(3), 141–169. https://doi.org/10.1002/per.2410010304

Lee, D., Henderson, A., &Shum, D. (2004). The effect of music on preprocedure anxiety in Hong Kong Chinese day patients. *Journal of Clinical Nursing*, *13*(3), 297–303. https://doi.org/10.1046/j.1365-2702.2003.00888.x

McCann, M. E., &Kain, Z. N. (2001). The Management of Preoperative Anxiety in Children: An Update. *Anesthesia and Analgesia*, *93*(1), 98–105. https://doi.org/10.1097/00000539-200107000-00022

Messina, M., Molinaro, F., Meucci, D., Angotti, R., Giuntini, L., Cerchia, E., Bulotta, A. L., &Brandigi, E. (2014). Preoperative distraction in children: hand-held videogames vs

clown therapy. *La Pediatria Medica e Chirurgica : Medical and Surgical Pediatrics, 36*(5–6), 98. https://doi.org/10.4081/pmc.2014.98

Mitchell, M. (2003). Patient anxiety and modern elective surgery: A literature review. *Journal of Clinical Nursing, 12*(6), 806–815. https://doi.org/10.1046/j.1365-2702.2003.00812.x

Moerman, N., vanDam, F. S., Muller, M. J., &Oosting, H. (1996). The Amsterdam Preoperative Anxiety and Information Scale (APAIS). *Anesthesia and Analgesia, 82*(3), 445–451. https://doi.org/10.1097/00000539-199603000-00002

Moher, D., Liberati, A., Tetzlaff, J., Altman, D. G., Altman, D. G., Antes, G., Atkins, D., Barbour, V., Barrowman, N., Berlin, J. A., Clark, J., Clarke, M., Cook, D., D'Amico, R., Deeks, J. J., Devereaux, P. J., Dickersin, K., Egger, M., Ernst, E., …Tugwell, P. (2009). Preferred reporting items for systematic reviews and meta-analyses: The PRISMA statement. In *PLoS Medicine*. https://doi.org/10.1371/journal.pmed.1000097

Ng, T. Y. (2002). *Sense-of-presence in virtual and real environments showing similar content: Questionnaire development and relationships among sense-of-presence, performance and cybersickness.* Hong Kong University of Science and Technology.

Niknejad, R., Mirmohammad-Sadeghi, M., Akbari, M., &Ghadami, A. (2019). Effects of an orientation tour on preoperative anxiety in candidates for coronary artery bypass grafting: A randomized clinical trial. *ARYA Atherosclerosis, 15*(4), 154–160. https://doi.org/10.22122/arya.v15i4.1806

Nintendo. (2019). *Technical Specs*. https://www.nintendo.com/switch/tech-specs/

Noben, L., Goossens, S. M. T. A., Truijens, S. E. M., VanBerckel, M. M. G., Perquin, C. W., Slooter, G. D., &VanRooijen, S. J. (2019). A virtual reality video to improve information provision and reduce anxiety before cesarean delivery: Randomized

controlled trial. *Journal of Medical Internet Research*, *21*(12), 1–12. https://doi.org/10.2196/15872

Parsons, T. D., Rizzo, A. A., Rogers, S., &York, P. (2009). Virtual reality in paediatric rehabilitation: A review. In *Developmental Neurorehabilitation* (Vol. 12, Issue 4, pp. 224–238). https://doi.org/10.1080/17518420902991719

Perks, A., Chakravarti, S., &Manninen, P. (2009). Preoperative anxiety in neurosurgical patients. *Journal of Neurosurgical Anesthesiology*, *21*(2), 127–130. https://doi.org/10.1097/ANA.0b013e31819a6ca3

Ramos, M. I., Cardoso, M. J., Vaz, F., Torres, M. D., García, F., Blanco, G., &González, E. M. (2008). Influence of the grade of anxiety and level of cortisol on post-surgical recovery. *Actas Espanolas de Psiquiatria*, *36*(3), 133–137. http://www.ncbi.nlm.nih.gov/pubmed/18478452

Rasouli, M. R., Menendez, M. E., Sayadipour, A., Purtill, J. J., &Parvizi, J. (2016). Direct Cost and Complications Associated With Total Joint Arthroplasty in Patients With Preoperative Anxiety and Depression. *Journal of Arthroplasty*, *31*(2), 533–536. https://doi.org/10.1016/j.arth.2015.09.015

Rizzo, A. S., &Koenig, S. T. (2017). Is clinical virtual reality ready for primetime? *Neuropsychology*, *31*(8), 877–899. https://doi.org/10.1037/neu0000405

Robertson, A., Khan, R., Fick, D., Robertson, W. B., Gunaratne, R., Yapa, S., Bowden, V., Rajan, R., Gunaratne, D. R., Yapa, S., Bowden, V., Hoffman, H., &Rajan, R. (2017). The effect of Virtual Reality in reducing preoperative anxiety in patients prior to arthroscopic knee surgery: A randomised controlled trial. *2017 IEEE 5th International Conference on Serious Games and Applications for Health, SeGAH 2017*. https://doi.org/10.1109/SeGAH.2017.7939282

Ryu, J. H., Oh, A. Y., Yoo, H. J., Kim, J. H., Park, J. W., &Han, S. H. (2019). The effect of an immersive virtual reality tour of the operating theater on emergence delirium in children undergoing general anesthesia: A randomized controlled trial. *Paediatric Anaesthesia*, *29*(1), 98–105. https://doi.org/10.1111/pan.13535

Ryu, J. H., Park, J.-W., Nahm, F. S., Jeon, Y.-T., Oh, A.-Y., Lee, H. J., Kim, J.-H., &Han, S.-H. (2018). The Effect of Gamification through a Virtual Reality on Preoperative Anxiety in Pediatric Patients Undergoing General Anesthesia: A Prospective, Randomized, and Controlled Trial. *Journal of Clinical Medicine*, *7*(9), 284. https://doi.org/10.3390/jcm7090284

Ryu, J. H., Park, S. J., Park, J. W., Kim, J. W., Yoo, H. J., Kim, T. W., Hong, J. S., &Han, S. H. (2017). Randomized clinical trial of immersive virtual reality tour of the operating theatre in children before anaesthesia. *British Journal of Surgery, 104*(12), 1628–1633. https://doi.org/10.1002/bjs.10684

Samsung. (2019). *Samsung Gear VR with Controller*. https://www.samsung.com/global/galaxy/gear-vr/?cid=in_paid_affiliate_all_none_none_samsungeshop2020_banner_none_05012020

Sarah, S., &Lynn, S. (2019). Is the Amsterdam Preoperative Anxiety and Information Scale (APAIS) a Valid Tool in Guiding the Management of Preoperative Anxiety in Adult Patients? A Literature Review. *Journal of Nursing and Practice, 3*(1), 95–102. https://doi.org/10.36959/545/368

Shahrbanian, S., Ma, X., Aghaei, N., Korner-Bitensky, N., Moshiri, K., &Simmonds, M. J. (2012). Use of virtual reality (immersive vs. non immersive) for pain management in children and adults: A systematic review of evidence from randomized controlled trials. In *Pelagia Research Library European Journal of Experimental Biology* (Issue 5). www.pelagiaresearchlibrary.com

Shri, R. (2010). *Anxiety : Causes and Management Symptoms of Anxiety Disorders*. *5*(1), 100–118.

Snaith, R. P. (2003). The hospital anxiety and depression scale. Health and Quality of Life Outcomes. *Health and Quality of Life Outcomes*, *10,* 1–11. https://doi.org/10.1186/1477-7525-1-29

Steimer, T. (2002). The biology of fear- and anxiety-related behaviors. *Dialogues in Clinical Neuroscience*, *4*(3), 231–249.

Suresh, K. (2011). An overview of randomization techniques: An unbiased assessment of outcome in clinical research. In *Journal of Human Reproductive Sciences* (Vol. 4, Issue 1, pp. 8–11). Wolters Kluwer -- Medknow Publications. https://doi.org/10.4103/0974-1208.82352

Turner-Cobb, J. M., &Hawken, T. (2019). Stress and Coping Assessment. In C. D.Llewellyn, C.McManus, J.Weinman, K. J.Petrie, S.Newman, S.Ayers, &T. A.Revenson (Eds.), *Cambridge Handbook of Psychology, Health and Medicine* (3rd ed., pp. 229–236). Cambridge University Press. https://doi.org/DOI: undefined

Wahn, B., &König, P. (2017). Is attentional resource allocation across sensory modalities task-dependent? *Advances in Cognitive Psychology*, *13*(1), 83–96. https://doi.org/10.5709/acp-0209-2

Wiens, A. G. (1998). Preoperative anxiety in women. *AORN Journal*, *68*(1). https://doi.org/10.1016/S0001-2092(06)62716-6

Wong, E. M. L., Chan, S. W. C., &Chair, S. Y. (2010). Effectiveness of an educational intervention on levels of pain, anxiety and self-efficacy for patients with musculoskeletal trauma. *Journal of Advanced Nursing*. https://doi.org/10.1111/j.1365-2648.2010.05273.x

Wu, H., Zhao, X., Chu, S., Xu, F., Song, J., Ma, Z., &Gu, X. (2020). Validation of the Chinese version of the Amsterdam Preoperative Anxiety and Information Scale (APAIS). *Health and Quality of Life Outcomes*, *18*(1), 1–6. https://doi.org/10.1186/s12955-020-01294-3

Yang, J.-H. H., Ryu, J. J., Nam, E., Lee, H.-S. S., &Lee, J. K. (2019). Effects of Preoperative Virtual Reality Magnetic Resonance Imaging on Preoperative Anxiety in Patients Undergoing Arthroscopic Knee Surgery: A Randomized Controlled Study. *Arthroscopy - Journal of Arthroscopic and Related Surgery*, *35*(8), 2394–2399. https://doi.org/10.1016/j.arthro.2019.02.037

Yeh, S. C., Li, Y. Y., Zhou, C., Chiu, P. H., &Chen, J. W. (2018). Effects of Virtual Reality and Augmented Reality on Induced Anxiety. *IEEE Transactions on Neural Systems and Rehabilitation Engineering*, *26*(7), 1345–1352. https://doi.org/10.1109/TNSRE.2018.2844083

個人資料 (第一版 21/06/2021)

(由研究員填寫)

請提供你的個人資料，所有資料絕對保密，只作研究用途。

參加者編號	______________________________	
1.	年齡	_________________
2.	性別	☐ 男　☐ 女
3.	婚姻狀況	☐ 未婚<br>☐ 已婚<br>☐ 離婚/分居<br>☐ 喪偶
4.	教育程度	☐ 小學或以下<br>☐ 中學<br>☐ 大學/專上教育
5.	居住狀況<br>（可選擇多於一個答案）	☐ 獨居<br>☐ 與配偶同住<br>☐ 與子女同住<br>☐ 與傭人同住<br>☐ 其他 ______________________________
	就業狀況	☐ 就業<br>☐ 失業/待業<br>☐ 料理家務<br>☐ 學生<br>☐ 退休<br>☐ 其他 ___________________________
7.	在此之前，你有否曾做過手術的經驗?	☐ 有　☐ 沒有
8.	在此之前，你有否曾入住醫院的經驗?	☐ 有　☐ 沒有
9.	美國麻醉師協會(ASA) 評分	☐ I 級<br>☐ II 級<br>☐ III 級<br>☐ VI 級<br>☐ V 級

Demographic Data Sheet (Version 1, 21/06/2021)

(To be completed by Research Team)

Please provide your information. All information will be kept confidential and be used for this study only.

**Subject Code**	**____________________________**	
**1.**	**Age**	**________________**
**2.**	**Gender**	☐ **Male** ☐ **Female**
**3.**	**Marital Status**	☐ **Single**<br>☐ **Married**<br>☐ **Divorced/Separated**<br>☐ **Widow**
**4.**	**Education Level**	☐ **Primary or below**<br>☐ **Secondary**<br>☐ **University / Higher Education**
**5.**	**Living Status**<br>**(Multiple selection if applicable)**	☐ **Living Alone**<br>☐ **Living with Partner**<br>☐ **Living with Sibling**<br>☐ **Living with Maid**<br>☐ **Other** **_________________________**
**6.**	**Employment Status**	☐ **Employed**<br>☐ **Unemployed / Between Jobs**<br>☐ **Housekeeper**<br>☐ **Student**<br>☐ **Retired**<br>☐ **Other** **_________________________**
**7.**	**Before this episode, do you have any previous experience of surgery?**	☐ **Yes** ☐ **No**
**8.**	**Before this episode, do you have any previous experience of admission to hospital?**	☐ **Yes** ☐ **No**
**9.**	**American Society of Anesthesiologists Classification (ASA) Grading**	☐ **Grade I**<br>☐ **Grade II**<br>☐ **Grade III**<br>☐ **Grade VI**<br>☐ **Grade V**

資料收集問卷 – T0 (第一頁) (第一版 21/06/2021)

(由研究員填寫)

所有資料絕對保密，只作研究用途。

參加者編號	______________________

1. 阿姆斯特丹術前焦慮與信息量表

Amsterdam Preoperative Anxiety and Information Scale (APAIS)

(Moerman et al., 1996)

問題	完全沒有				非常明顯
	1	2	3	4	5
1. 我對麻醉感到擔心					
2. 我一直在想麻醉這件事					
3. 我希望盡可能多地了解有關麻醉的事					
4. 我對手術感到擔心					
5. 我一直在想手術這件事					
6. 我希望盡可能多地了解有關手術的事					

資料收集問卷 – T0 (第二頁) (第一版 21/06/2021)

(由研究員填寫)

所有資料絕對保密，只作研究用途。

2. 虛擬實境暈眩量表

Simulation Sickness Questionnaire (SSQ)

(Kennedy et al, 1993; Huang, 2013)

請依照目前的身體狀態，在以下 16 項不適症狀之輕重程度上打圈。

編號	症狀	症狀程度			
1.	一般不適感	無症狀	輕微症狀	中等症狀	嚴重症狀
2.	疲勞	無症狀	輕微症狀	中等症狀	嚴重症狀
3.	頭痛	無症狀	輕微症狀	中等症狀	嚴重症狀
4.	眼睛疲勞	無症狀	輕微症狀	中等症狀	嚴重症狀
5.	聚焦困難	無症狀	輕微症狀	中等症狀	嚴重症狀
6.	唾液分泌增加	無症狀	輕微症狀	中等症狀	嚴重症狀
7.	出汗	無症狀	輕微症狀	中等症狀	嚴重症狀
8.	噁心	無症狀	輕微症狀	中等症狀	嚴重症狀
9.	難以集中	無症狀	輕微症狀	中等症狀	嚴重症狀
10.	頭腦發漲	無症狀	輕微症狀	中等症狀	嚴重症狀
11.	視力模糊	無症狀	輕微症狀	中等症狀	嚴重症狀
12.	睜眼時目眩	無症狀	輕微症狀	中等症狀	嚴重症狀
13.	閉眼時目眩	無症狀	輕微症狀	中等症狀	嚴重症狀
14.	眩暈	無症狀	輕微症狀	中等症狀	嚴重症狀
15.	胃部不適	無症狀	輕微症狀	中等症狀	嚴重症狀
16.	打嗝	無症狀	輕微症狀	中等症狀	嚴重症狀

資料收集問卷 – T0 (第三頁) (第一版 21/06/2021)

(由研究員填寫)

所有資料絕對保密，只作研究用途。

3. 壓力評估

Stress Level

請在這直線上垂直畫「一短線」，以代表你**目前**的壓力程度，0 毫米代表「無壓力」，100 毫米代表「極大壓力」。

0 毫米
無壓力

100 毫米
極大壓力

4. 預備度評估

Preparedness Level

請在這直線上垂直畫「一短線」，以代表你**目前**對於手術的預備度，0 毫米代表「沒有預備」，100 毫米代表「準備充足」。

0 毫米
沒有預備

100 毫米
準備充足

資料收集問卷 – T1 (第一頁) (第一版 21/06/2021)

(由研究員填寫)

所有資料絕對保密，只作研究用途。

參加者編號	______________________

1. 阿姆斯特丹術前焦慮與信息量表

Amsterdam Preoperative Anxiety and Information Scale (APAIS)

(Moerman et al., 1996)

問題	完全沒有				非常明顯
	1	2	3	4	5
1. 我對麻醉感到擔心					
2. 我一直在想麻醉這件事					
3. 我希望盡可能多地了解有關麻醉的事					
4. 我對手術感到擔心					
5. 我一直在想手術這件事					
6. 我希望盡可能多地了解有關手術的事					

資料收集問卷 – T1 (第二頁) (第一版 21/06/2021)

(由研究員填寫)

所有資料絕對保密，只作研究用途。

2. 虛擬實境暈眩量表

Simulation Sickness Questionnaire (SSQ)

(Kennedy et al, 1993; Huang, 2013)

請依照目前的身體狀態，在以下 16 項不適症狀之輕重程度上打圈。

編號	症狀	症狀程度			
1.	一般不適感	無症狀	輕微症狀	中等症狀	嚴重症狀
2.	疲勞	無症狀	輕微症狀	中等症狀	嚴重症狀
3.	頭痛	無症狀	輕微症狀	中等症狀	嚴重症狀
4.	眼睛疲勞	無症狀	輕微症狀	中等症狀	嚴重症狀
5.	聚焦困難	無症狀	輕微症狀	中等症狀	嚴重症狀
6.	唾液分泌增加	無症狀	輕微症狀	中等症狀	嚴重症狀
7.	出汗	無症狀	輕微症狀	中等症狀	嚴重症狀
8.	噁心	無症狀	輕微症狀	中等症狀	嚴重症狀
9.	難以集中	無症狀	輕微症狀	中等症狀	嚴重症狀
10.	頭腦發漲	無症狀	輕微症狀	中等症狀	嚴重症狀
11.	視力模糊	無症狀	輕微症狀	中等症狀	嚴重症狀
12.	睜眼時目眩	無症狀	輕微症狀	中等症狀	嚴重症狀
13.	閉眼時目眩	無症狀	輕微症狀	中等症狀	嚴重症狀
14.	眩暈	無症狀	輕微症狀	中等症狀	嚴重症狀
15.	胃部不適	無症狀	輕微症狀	中等症狀	嚴重症狀
16.	打嗝	無症狀	輕微症狀	中等症狀	嚴重症狀

資料收集問卷 – T1 (第三頁) (第一版 21/06/2021)

(由研究員填寫)

所有資料絕對保密，只作研究用途。

3. 壓力評估

Stress Level

請在這直線上垂直畫「一短線」，以代表你**目前**的壓力程度，0 毫米代表「無壓力」，100 毫米代表「極大壓力」。

0 毫米
無壓力

100 毫米
極大壓力

4. 預備度評估

Preparedness Level

請在這直線上垂直畫「一短線」，以代表你**目前**對於手術的預備度，0 毫米代表「沒有預備」，100 毫米代表「準備充足」。

0 毫米
沒有預備

100 毫米
準備充足

5. 疼痛評估

Pain Level

請在這直線上垂直畫「一短線」，以代表你**目前**的疼痛程度，0 毫米代表「沒有痛楚」，100 毫米代表「極大痛楚」。

0 毫米
沒有痛楚

100 毫米
極大痛楚

資料收集問卷 – T2 (第一頁) (第一版 21/06/2021)

(由研究員填寫)

所有資料絕對保密，只作研究用途。

參加者編號	________________________

1. 阿姆斯特丹術前焦慮與信息量表
Amsterdam Preoperative Anxiety and Information Scale (APAIS)

(Moerman et al., 1996)

問題	完全沒有				非常明顯
	1	2	3	4	5
1. 我對麻醉感到擔心					
2. 我一直在想麻醉這件事					
3. 我希望盡可能多地了解有關麻醉的事					
4. 我對手術感到擔心					
5. 我一直在想手術這件事					
6. 我希望盡可能多地了解有關手術的事					

資料收集問卷 – T2 (第二頁) (第一版 21/06/2021)

(由研究員填寫)

所有資料絕對保密，只作研究用途。

2. 壓力評估

Stress Level

請在這直線上垂直畫「一短線」，以代表你**目前**的壓力程度，0 毫米代表「無壓力」，100 毫米代表「極大壓力」。

0 毫米 無壓力 —— 100 毫米 極大壓力

3. 預備度評估

Preparedness Level

請在這直線上垂直畫「一短線」，以代表你**目前**對於手術的預備度，0 毫米代表「沒有預備」，100 毫米代表「準備充足」。

0 毫米 沒有預備 —— 100 毫米 準備充足

4. 疼痛評估

Pain Level

請在這直線上垂直畫「一短線」，以代表你**目前**的疼痛程度，0 毫米代表「沒有痛楚」，100 毫米代表「極大痛楚」。

0 毫米 沒有痛楚 —— 100 毫米 極大痛楚

資料收集問卷 – T3 (第一頁) (第一版 21/06/2021)

(由研究員填寫)

所有資料絕對保密，只作研究用途。

參加者編號	______________________

1. 疼痛評估

Pain Level

請在這直線上垂直畫「一短線」，以代表你**目前**的疼痛程度，0 毫米代表「沒有痛楚」，100 毫米代表「極大痛楚」。

0 毫米
沒有痛楚

100 毫米
極大痛楚

2. 服務評估

Satisfaction Level

請在這直線上垂直畫「一短線」，以代表你**對於**術前服務的滿意程度，0 毫米代表「極不滿意」，100 毫米代表「非常滿意」。

0 毫米
極不滿意

100 毫米
非常滿意

Data Collection Questionnaire – T0 (Page 1) (ver1: 21/06/2021)

(To be completed by Researcher)

All information will be kept confidential and be used for this study only.

**Subject Code**	____________________________

1. Amsterdam Preoperative Anxiety and Information Scale (APAIS)

(Moerman et al., 1996)

Question	Not at all				Very much
	1	2	3	4	5
1. I am worried about the anaesthetic					
2. The anesthetic is on my mind continually					
3. I would like to know as much as possible about the anesthetic					
4. I am worried about the procedure					
5. The procedure is on my mind continually					
6. I would like to know as much as possible about the procedure					

Data Collection Questionnaire – T0 (Page 2) (ver1: 21/06/2021)

(To be completed by Researcher)

All information will be kept confidential and be used for this study only.

2. Simulation Sickness Questionnaire (SSQ)

(Kennedy et al, 1993; Huang, 2013)

Instructions: Circle how much each symptom below is affecting you right now.

No.	Symptoms	Level of Symptoms			
1.	General discomfort	None	Slight	Moderate	Severe
2.	Fatigue	None	Slight	Moderate	Severe
3.	Headache	None	Slight	Moderate	Severe
4.	Eyestrain	None	Slight	Moderate	Severe
5.	Difficulty focusing	None	Slight	Moderate	Severe
6.	Salivation increase	None	Slight	Moderate	Severe
7.	Sweating	None	Slight	Moderate	Severe
8.	Nausea	None	Slight	Moderate	Severe
9.	Difficulty concentrating	None	Slight	Moderate	Severe
10.	Fullness of the head	None	Slight	Moderate	Severe
11.	Blurred vision	None	Slight	Moderate	Severe
12.	Dizziness with eyes open	None	Slight	Moderate	Severe
13.	Dizzy (eyes close)	None	Slight	Moderate	Severe
14.	Vertigo	None	Slight	Moderate	Severe
15.	Stomach awareness	None	Slight	Moderate	Severe
16.	Burping	None	Slight	Moderate	Severe

Data Collection Questionnaire – T0 (Page 3) (ver1: 21/06/2021)

(To be completed by Researcher)

All information will be kept confidential and be used for this study only.

3. Stress Level – Visual Analogue Scale

Please draw a line on the above scale to indicate your current stress level, while 0 mm indicates you have "No Pressure" and 100 mm indicates you have "Extremely Stressful".

0 mm
No Stress

100 mm
Extremely Stressful

4. Preparedness Level – Visual Analogue Scale

Please draw a line on the above scale to indicate your current preparedness level, while 0 mm indicates you have "Not Prepared" and 100 mm indicates you have "Well Prepared".

0 mm
Not Prepared

100 mm
Well Prepared

Data Collection Questionnaire – T1 (Page 1) (ver1: 21/06/2021)

(To be completed by Researcher)

All information will be kept confidential and be used for this study only.

**Subject Code**	______________________________

1. Amsterdam Preoperative Anxiety and Information Scale (APAIS)

(Moerman et al., 1996)

Question	Not at all				Very much
	1	2	3	4	5
7. I am worried about the anaesthetic					
8. The anesthetic is on my mind continually					
9. I would like to know as much as possible about the anesthetic					
10. I am worried about the procedure					
11. The procedure is on my mind continually					
12. I would like to know as much as possible about the procedure					

Data Collection Questionnaire – T1 (Page 2) (ver1: 21/06/2021)

(To be completed by Researcher)

All information will be kept confidential and be used for this study only.

2. Simulation Sickness Questionnaire (SSQ)

(Kennedy et al, 1993; Huang, 2013)

Instructions: Circle how much each symptom below is affecting you right now.

No.	Symptoms	Level of Symptoms			
1.	General discomfort	None	Slight	Moderate	Severe
2.	Fatigue	None	Slight	Moderate	Severe
3.	Headache	None	Slight	Moderate	Severe
4.	Eyestrain	None	Slight	Moderate	Severe
5.	Difficulty focusing	None	Slight	Moderate	Severe
6.	Salivation increase	None	Slight	Moderate	Severe
7.	Sweating	None	Slight	Moderate	Severe
8.	Nausea	None	Slight	Moderate	Severe
9.	Difficulty concentrating	None	Slight	Moderate	Severe
10.	Fullness of the head	None	Slight	Moderate	Severe
11.	Blurred vision	None	Slight	Moderate	Severe
12.	Dizziness with eyes open	None	Slight	Moderate	Severe
13.	Dizzy (eyes close)	None	Slight	Moderate	Severe
14.	Vertigo	None	Slight	Moderate	Severe
15.	Stomach awareness	None	Slight	Moderate	Severe
16.	Burping	None	Slight	Moderate	Severe

Data Collection Questionnaire – T1 (Page 3) (ver1: 21/06/2021)

(To be completed by Researcher)

All information will be kept confidential and be used for this study only.

3. Stress Level – Visual Analogue Scale

Please draw a line on the above scale to indicate your current stress level, while 0 mm indicates you have “No Pressure” and 100 mm indicates you have “Extremely Stressful”.

0 mm
No Stress

100 mm
Extremely Stressful

4. Preparedness Level – Visual Analogue Scale

Please draw a line on the above scale to indicate your current preparedness level, while 0 mm indicates you have “Not Prepared” and 100 mm indicates you have “Well Prepared”.

0 mm
Not Prepared

100 mm
Well Prepared

5. Pain Level – Visual Analogue Scale

Please draw a line on the above scale to indicate your current pain level, while 0 mm indicates you have “No Pain” and 100 mm indicates you have “Worst Pain”.

0 mm
No Pain

100 mm
Worst Pain

Data Collection Questionnaire – T2 (Page 1) (ver1: 21/06/2021)

(To be completed by Researcher)

All information will be kept confidential and be used for this study only.

**Subject Code**	______________________________

1. Amsterdam Preoperative Anxiety and Information Scale (APAIS)

(Moerman et al., 1996)

Question	Not at all				Very much
	1	2	3	4	5
13. I am worried about the anaesthetic					
14. The anesthetic is on my mind continually					
15. I would like to know as much as possible about the anesthetic					
16. I am worried about the procedure					
17. The procedure is on my mind continually					
18. I would like to know as much as possible about the procedure					

Data Collection Questionnaire – T2 (Page 2) (ver1: 21/06/2021)

(To be completed by Researcher)

All information will be kept confidential and be used for this study only.

2. Stress Level – Visual Analogue Scale

Please draw a line on the above scale to indicate your <u>current</u> stress level, while 0 mm indicates you have “No Pressure” and 100 mm indicates you have “Extremely Stressful”.

0 mm
No Stress

100 mm
Extremely Stressful

3. Preparedness Level – Visual Analogue Scale

Please draw a line on the above scale to indicate your <u>current</u> preparedness level, while 0 mm indicates you have “Not Prepared” and 100 mm indicates you have “Well Prepared”.

0 mm
Not Prepared

100 mm
Well Prepared

4. Pain Level – Visual Analogue Scale

Please draw a line on the above scale to indicate your <u>current</u> pain level, while 0 mm indicates you have “No Pain” and 100 mm indicates you have “Worst Pain”.

0 mm
No Pain

100 mm
Worst Pain

Data Collection Questionnaire – T3 (Page 1) (ver1: 21/06/2021)

(To be completed by Researcher)

All information will be kept confidential and be used for this study only.

**Subject Code**	________________________

1. Pain Level – Visual Analogue Scale

Please draw a line on the above scale to indicate your current pain level, while 0 mm indicates you have “No Pain” and 100 mm indicates you have “Worst Pain”.

0 mm
No Pain

100 mm
Worst Pain

2. Satisfaction Level – Visual Analogue Scale

Please draw a line on the above scale to indicate your overall satisfaction level, while 0 mm indicates you have “Not Prepared” and 100 mm indicates you have “Well Prepared”.

0 mm
Poorly Satisfy

100 mm
Extremely Satisfy

香港中文大學那打素護理學院
參與者資料頁
(第 1.1 版, 2021 年 9 月 15 日)

研究題目

虛擬現實為本的措施對於減低接受非緊急手術的成年病人手術前焦慮的影響之隨機對照試驗

現在我們邀請你參與一項研究計劃。在你還沒有決定是否參與之前，請務必瞭解研究的目的及研究將涉及什麼。這份資料書詳述有關資訊，請仔細閱讀以下資料。如有必要，請你和你的家人、朋友及你的醫生討論。若你有任何疑問，或想知道更多的資料，請向負責這項研究的人員詢問，然後周詳考慮並決定是否參與。

研究背景

大多數病人對手術會感到壓力，並且通常與焦慮有關。亦有可能因此引致廣泛的生理、情緒和認知上的負面影響，並影響手術結果。儘管術前焦慮在臨床中很常見，但它經常被忽略，並誤以為正常反應。現今臨床上應用傳統藥物和非藥物措施減低術前焦慮方面存在很多限制。文獻指出虛擬現實為本的措施可以有效減低術前焦慮，但當中虛擬現實為本的措施加上術前教育對成年人之成效尚未確定。因目前還沒有本地關於虛擬現實為本的措施對減少術前焦慮（包括制定到實施）的影響之研究，本研究希可以確定在本地環境中使用虛擬現實為本的措施來減少術前焦慮的效果和可行性。

研究目的

此項研究目的是設計和開發為減低術前焦慮的虛擬現實為本措施，並評估虛擬現實為本的措施對於減低接受手術的病人手術前焦慮的影響。

研究程式

您被邀請參加這項研究是因為您在術前評估診所為非緊急手術接受檢查。大約 90 位參與者將會參加這項研究。為了找出醫治病人的最佳方法，有時我們需要把不同的治病方法作比較。我們會把參與病人用電腦以隨機的方式分組，而參與病人的個別資料並不會輸入電腦。在這研究中，你不會知道自己被分配接受何種治療。虛擬現實為本的措施或一般治療不同組別的病人將會接受不同的治療，並把所得的結果作比較，需時約 10-15 分鐘。。

參與的利弊

本研究之措施將會利用一體式虛擬現實眼鏡實行。然而，我們的研究小組無法保證你會因為參與此項研究而得到任何直接醫療益處。從這項研究中獲得的資料可能會在將來幫助其他病人。從這項研究中獲得的資料可能會幫助醫護人員更深入明了解虛擬現實為本的措施之效果，從而找出更合適的治療方法去治理相關問題。

參與研究收費及報酬

除要繳付常規醫療費用外，您無需繳交額外費用及不會收到任何報酬。

治療和賠償
如研究人員察覺您在參與過程中，情緒或身體上有任何不適現象，研究人員將會為您進行治療或轉介您接受治療，您亦可以隨時中止。您不會透過簽署本同意書而放棄任何法律權利。

新資料
在研究進行期間，如果有重要的新資訊出現，而這資訊可能會影響你繼續參與研究的意願，研究人員將會盡快通知你，並與你討論是否讓你繼續參與。若你決定繼續參與，我們將要求你簽署一份修訂的同意書。若你決定退出，研究人員會安排讓你繼續得到照顧。另外，如研究人員認為終止你的參與比較適當，他／她會向你解釋你不能繼續參與的原因並為你安排往後的治療。

自動參與/中途退出/終止研究
根據經驗，這項研究對參與者並不會帶來不適或危險。你的參與純粹為自願性質。你可以隨時選擇不參加或是退出這項研究，而不會影響你現時接受的治療。你可以隨時對研究提出問題。一旦您要退出研究，如果沒有得到您的同意，退出前所收集的數據將會被銷毀。您亦可於知情同意書表示允許研究人員在您退出後繼續使用從您身上所收集的數據用於本研究用途。若有需要，亦可以直接聯絡研究人員。此項研究及研究協議已獲新界西醫院聯網研究倫理委員會和香港中文大學 – 新界東醫院聯網臨床研究倫理聯席委員會批核。

保密原則
所有研究的資料不會記名及絕對保密，只作分析用途，所有問卷資料將存放於一個鎖上的檔櫃內，在研究完成 5 年後，所有資料一律會被銷毀。如你決定參加這項研究，研究小組將會使用關於你的健康資料來進行這項研究。亦可能會與負責檢查這項研究的倫理委員會分享你的健康資料以確保研究規定被正確遵守。從這項研究得出的資料可能會刊載於國際醫學期刊或在會議發表，但參加者的姓名將會保密。

根據香港法律規定（特別是第 486 章《個人資料（私隱）條例》），您享有或可享有確保您的個人資料保密的權利，例如在或為本研究中有關收集、監管、保留、管理、控制、使用（包括分析或比較）、轉進或轉出香港、不披露、清除和／或以任何方式處理或棄置的權利。如有任何問題，請您諮詢個人資料私隱專員或其職員（電話號碼：2827 2827），以瞭解妥善監控或監管您的個人資料保護之事宜，以確保您完整掌握和瞭解遵守規管個人資料私隱的法律之重要性。

問題查詢
此項研究由趙柏龍註冊護士（中文大學那打素護理學院護理學博士學生）執行，並由黃祖莉教授 (香港中文大學那打素護理學院，聯絡電話：3943 8166) 監督。如有任何查詢關於此項研究，歡迎致電 9463 0241，聯絡本研究的主研究員趙柏龍先生。

若您對作為研究參加者所享有的權利有任何疑問，請致電 3767 7866 / 2468 6118 與新界西醫院聯網研究倫理委員會或 3505 3935 與香港中文大學-新界東醫院聯網臨床研究倫理聯席委員會聯絡。

誠邀閣下參加此項研究。

参加者編號:______________

香港中文大學那打素護理學院
知情同意書(第 1.1 版，2021 年 9 月 15 日)

研究題目: 虛擬現實為本的措施對於減低接受非緊急手術的成年病人手術前焦慮的影響之隨機對照試驗

研究員姓名: 趙柏龍

		請在方框中加 ✓
**1.**	我已閱讀及明白這份參與研究資料書 (____/____/____),並且已經獲得提問的權利。 日期	☐
**2.**	我明白我的參與完全出於自願並且可以在任何時候退出,而無需任何理由。我的決定不會影響我所受到的醫療待遇和法律權利。	☐
**3.**	我明白此研究的有關人員會查閱我的醫療記錄,我同意授權有關人員查閱我的記錄。	☐
**4.**	我同意參與這項研究。	☐

參與研究病人 ／監護人姓名	日期	簽名
*見證人姓名 (如適用)	與病人關係	簽名
*獲取同意者姓名<br>(如不是研究人員)	日期	簽名
研究人員姓名	日期	簽名

如有任何有關這項研究的問題或緊急情況,請電與趙柏龍先生 (9463 0241) 或 黃祖莉教授 (3943 8166) 聯絡。

副本致: (1) 參與研究病人/監護人 (2) 研究人員檔案 (3) 醫院檔案

***〔請刪去不適用者〕**

The Chinese University of Hong Kong
The Nethersole School of Nursing

Information sheet for participants

(Version 1.1, 14 September 2021)

Research Topic

Effects of virtual reality based intervention on reducing preoperative anxiety among patients undergoing elective surgery: a randomized controlled trial

You are invited to take part in this research. Before you decide to participate it is important for you to understand why the research is being done and what it will involve. Please take time to read the following information carefully and discuss it with friends, relatives and your doctor if you wish. Ask us if there is anything that is not clear and/or if you would like more information. Take time to decide whether or not you wish to take part in this research.

The study background

Surgery is a stressful experience to most of the patients and commonly associated with anxiety. A wide range of negative consequences, including physiological, emotional and cognitive effects could be resulted and affect the outcomes of the surgery. Although preoperative anxiety is common in clinical setting, it often accepted negligently as a normal response. While limitations existed in the traditional pharmacological and non-pharmacological intervention for managing preoperative anxiety, evidence demonstrated virtual reality (VR) based intervention could effectively manage preoperative anxiety and it is still uncertain that if VR based intervention with preoperative education could be benefit to adult population. To the best knowledge, there was no local study conducted in evaluating VR based intervention, from development to implementation, and effect in reducing preoperative anxiety. The study could identify the effect and feasibility on reducing preoperative anxiety via VR based intervention in local setting.

Purpose of study

The objectives of this study are to design and develop the VR based intervention for managing preoperative anxiety and to evaluate the effectiveness of the VR based intervention to reduce preoperative anxiety among adult patients undergoing surgery.

Procedure

Patients attend the pre-anaesthetic assessment clinic for scheduled elective surgery undergoing general anaesthesia and consented to the study will be invited to participate in this study. Around 90 participants will be invited to join this study. Sometimes because we need to know which way of treating patients is the best, we need to make comparisons. Study participants will be put into groups and then compared. The study participants are randomly selected by a computer which has no distinguishing information about the individual, i.e. by chance. Patients in each group will then have a different treatment and these are compared. In a blind trial you will not know which treatment group you are in. Intervention group will receive VR based intervention and control group received usual standard care. Both groups of participants will receive assessment for data collection before and after intervention and surgery respectively in around 10 minutes.

Risk and benefit

The intervention is conducted by all-in-one VR console. It will not cause any pain, discomfort, or harm to you, while the potential benefit is to reduce preoperative anxiety. We hope the treatment may help you. However, it cannot be guaranteed. The information we get from this research may help us treat future patients with preoperative anxiety better.

Cost and Payment of the Study

Apart from paying standard hospital or clinic fees, you are not required to pay additional fees and will not receive any remuneration.

Treatment and Compensation

If you feel uncomfortable emotionally or physically during the intervention, you could stop the intervention at any time and researchers will provide support to you or refer you to have appropriate treatment. You are not giving up any of legal rights by signing this form.

New Information

Our research team will inform you if significant new information about the intervention under research is available. You may decide whether to continue to participate in the research or not. If you decide to continue, you will be asked to sign an updated consent form. If you choose to withdraw, research team will arrange for your continued care. On the other hand, the research team may suspend your participation in the research should he/she consider it to be in your best interest. He/she will explain to you and arrange for your continued care.

Voluntary participation/ Withdrawal / Termination

Participation in the study is voluntary. Your decision to participate or not will be respected. You have the right to terminate your participation at any time and without giving any reason during the study. If you feel uncomfortable in any way during the session, you may not continue to participate in the study. If you withdraw from the study, the data collected up to your withdrawal will not be used unless with your consent. You may also express your consent to research team through Informed Consent Form to allow research team to continuously use data collected before your withdrawal for research purpose. The study has been approved by New Territories West Cluster Research Ethics Committee (NTWC REC) and Joint Chinese University of Hong Kong – New Territories East Cluster Clinical Research Ethics Committee. If you have questions related to your rights as a research participant, please contact New Territories West Cluster Research Ethics Committee (NTWC REC) at 3767 7866 / 2468 6118 or the Joint Chinese University of Hong Kong-New Territories East Cluster Clinical Research Ethics Committee at 3505 3935.

Confidentiality

Anonymity and confidentiality of all information will always be maintained. The questionnaire will be kept in locked cabinet. The information will only be used for analytical purposes and will be destroyed at the end of the study. Under the laws of Hong Kong (in particular the Personal Data (Privacy) Ordinance, Cap 486), you enjoy or may enjoy rights for the protection of the confidentiality of your personal data, such as those regarding the collection, custody, retention, management, control, use (including analysis or comparison), transfer in or out of Hong Kong, non-disclosure, erasure and/or in any way dealing with or disposing of any of your personal data in or for this study. For any query, you should consult the Privacy Commissioner for Personal Data or his officer (Tel no.: 2827 2827) as to the proper monitoring or supervision of your personal data protection so that your full awareness and understanding of the significance of compliance with the law governing privacy data is assured. To supervise this study, you will authorize the ethics committee of the hospital (i.e. New Territories West Cluster Research Ethics Committee (NTWC REC)) to retrieve your personal data in accordance with the terms of this study and this consent form.

Inquiry

This study is conducted by Mr CHIU Pak Lung (Student of Doctor of Nursing, the Nethersole School of Nursing, the Chinese University of Hong Kong) and supervised by Professor WONG Cho Lee, Jojo (Assistant Professor, the Nethersole School of Nursing, the Chinese University of Hong

Kong). Should you have any questions about the study, please feel free to contact Mr. CHIU at 9463 0241 or Prof. WONG at 3943 8166.

If you have questions related to your rights as a research participant, please contact New Territories West Cluster Research Ethics Committee (NTWC REC) at 3767 7866 / 2468 6118 or the Joint Chinese University of Hong Kong- New Territories East Cluster Clinical Research Ethics Committee at 3505 3935.

You are cordially invited to participate in the study.

Subject Code:____________

The Chinese University of Hong Kong
The Nethersole School of Nursing

Patient/Subject Consent Form (Version 1.1, 14 September 2021)

Research Topic: Effect of virtual reality based intervention on reducing preoperative anxiety among patient undergoing surgery: a randomized controlled trial

Name of Researcher: CHIU Pak Lung

		Please initial box
**1.**	I confirm that I have read and understood the information sheet dated ___/___/___ for the above study and have had the opportunity to ask question.	☐
**2.**	I understand that my participation is voluntary and that I am free to withdraw at any time, without giving any reason, without my medical care or legal rights being affected.	☐
**3.**	I understand that my medical notes may be read by responsible individuals concerned in this research. I give permission for these individuals to have access to my records.	☐
**4.**	I agree to take part in the above study and to cooperate fully with the researcher.	☐

Name of Patient/Guardian	Date	Signature
Name of Witness (if applicable)	Relationship	Signature
Name of person taking consent (if different from researcher)	Date	Signature
Researcher	Date	Signature

In case of any emergency/any questions related to this study, please contact Mr. CHIU at 9463 0241 or Prof. WONG at 3943 8166.

Copies to: (1) Patient/Subject/Guardian (2) Researcher's File (3) Hospital Record
