# Supplementary material for: Virtual Reality–Based Intervention to Reduce Preoperative Anxiety in Adults Undergoing Elective Surgery: A Randomized Clinical Trial
Source: JAMA Netw Open. 2023 Oct 31;6(10):e2340588. doi: 10.1001/jamanetworkopen.2023.40588 (PMC10618840; doi:10.1001/jamanetworkopen.2023.40588)
Supplement: Supplement 2. — eFigure 1. Screen Captures of VR-Based Intervention Throughout the Entire Perioperative Process eFigure 2. Changes of Preoperative Anxiety Scores Across Three Time Points eFigure 3. Changes of Stress Level Across Three Time Points eFigure 4. Changes of Preparedness Across Three Time Points eFigure 5. Changes of Pain Level Across Four Time Points eFigure 6. Graphical Comparison of the Satisfaction of the Study Groups at T3 eFigure 7. Graphical Comparison of Postoperative Length of Stay Between Study Groups eAppendix. A Detailed Description of Each Measure Used in This Trial eReferences [file jamanetwopen-e2340588-s002.pdf]

Supplemental Online Content

Chiu PL, Li H, Yap KY, Lam KC, Yip PR, Wong CL. Virtual reality–based intervention to reduce preoperative anxiety in adults undergoing elective surgery: a randomized clinical trial. *JAMA Netw Open*. 2023;6(10):e2340588.
doi:10.1001/jamanetworkopen.2023.40588

eFigure 1. Screen Captures of VR-Based Intervention Throughout the Entire Perioperative Process

eFigure 2. Changes of Preoperative Anxiety Scores Across Three Time Points

eFigure 3. Changes of Stress Level Across Three Time Points

eFigure 4. Changes of Preparedness Across Three Time Points

eFigure 5. Changes of Pain Level Across Four Time Points

eFigure 6. Graphical Comparison of the Satisfaction of the Study Groups at T3

eFigure 7. Graphical Comparison of Postoperative Length of Stay Between Study Groups

eAppendix. A Detailed Description of Each Measure Used in This Trial

eReferences

This supplemental material has been provided by the authors to give readers additional information about their work.

© 2023 Chiu PL et al. *JAMA Network Open.*

eFigure 1. Screen Captures of VR-Based Intervention Throughout the Entire Perioperative Process

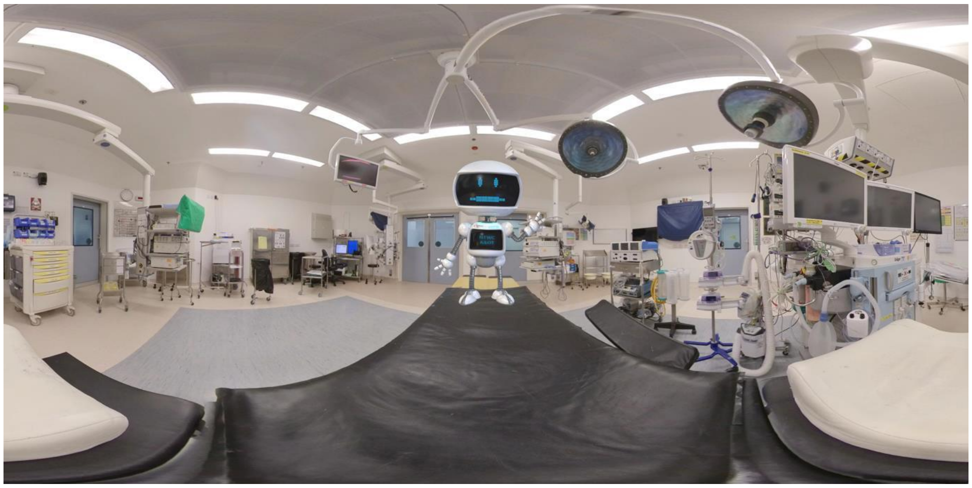

eFigure 1a. Scene 1: Introduction

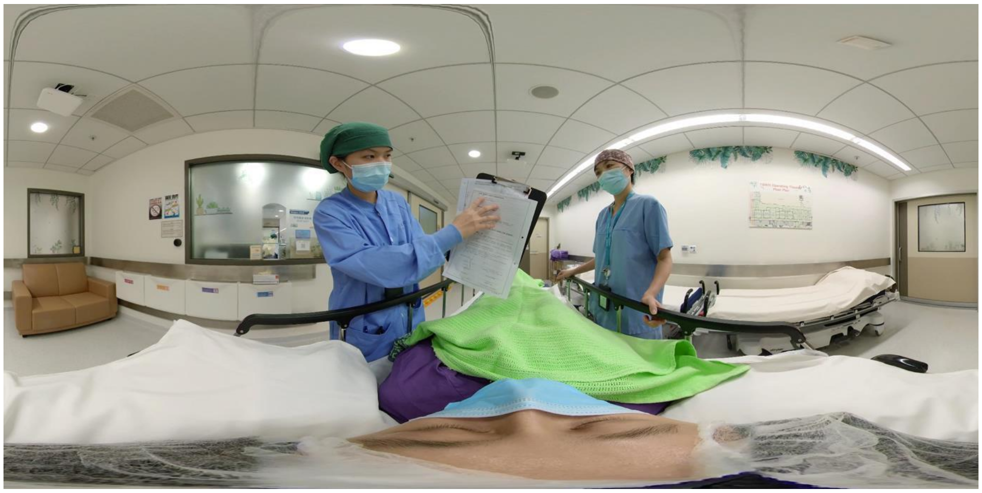

eFigure 1b. Scene 2: Reception

© 2023 Chiu PL et al. *JAMA Network Open.*

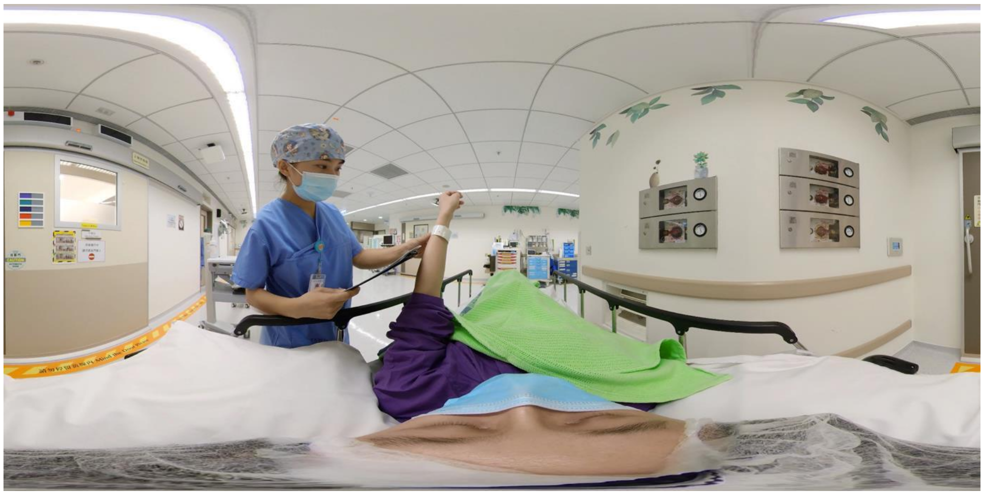

eFigure 1c. Scene 3: Induction

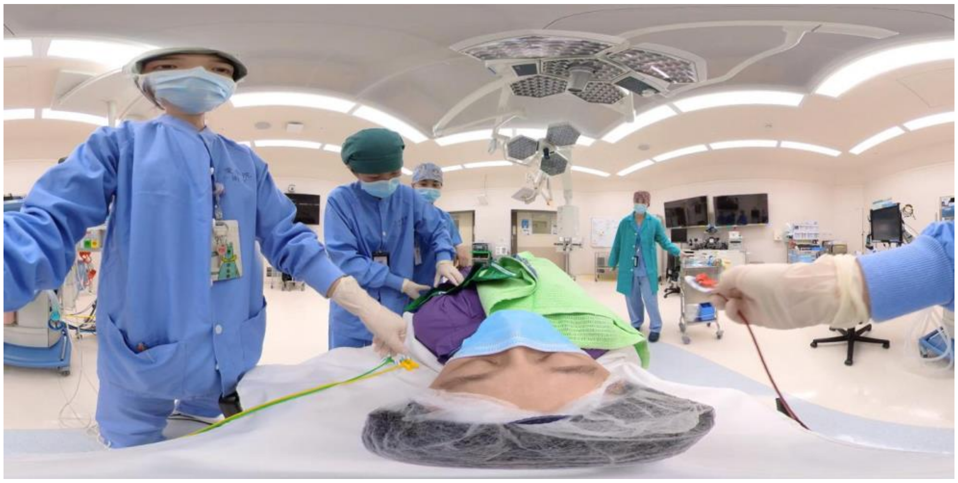

eFigure 1d. Scene 4: Operating Theatre

© 2023 Chiu PL et al. *JAMA Network Open.*

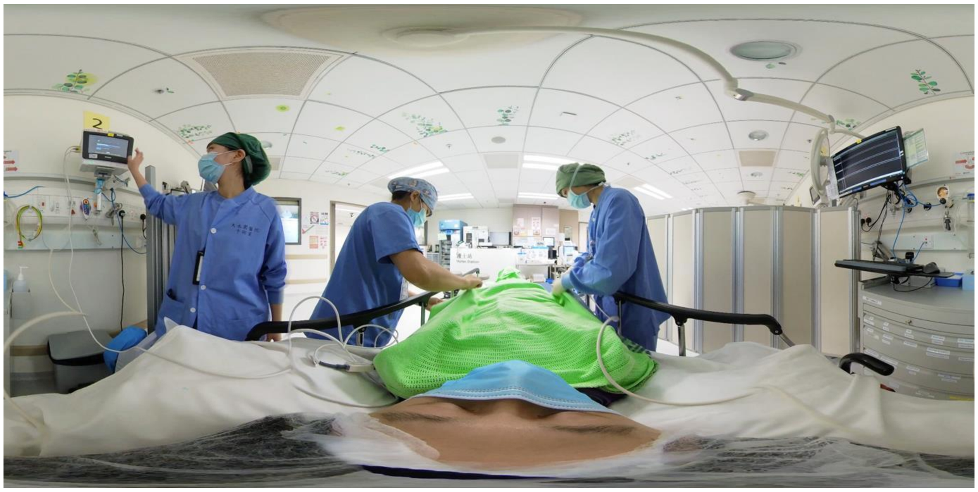

eFigure 1e. Scene 5: Post-anaesthesia care unit (PACU)

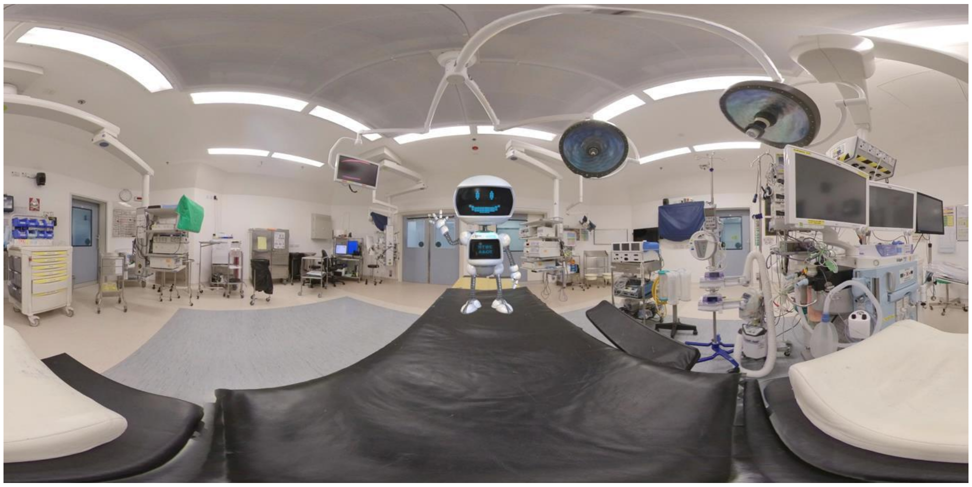

eFigure 1f. Scene 6: Ending

© 2023 Chiu PL et al. *JAMA Network Open.*

eFigure 2. Changes of Preoperative Anxiety Scores Across Three Time Points

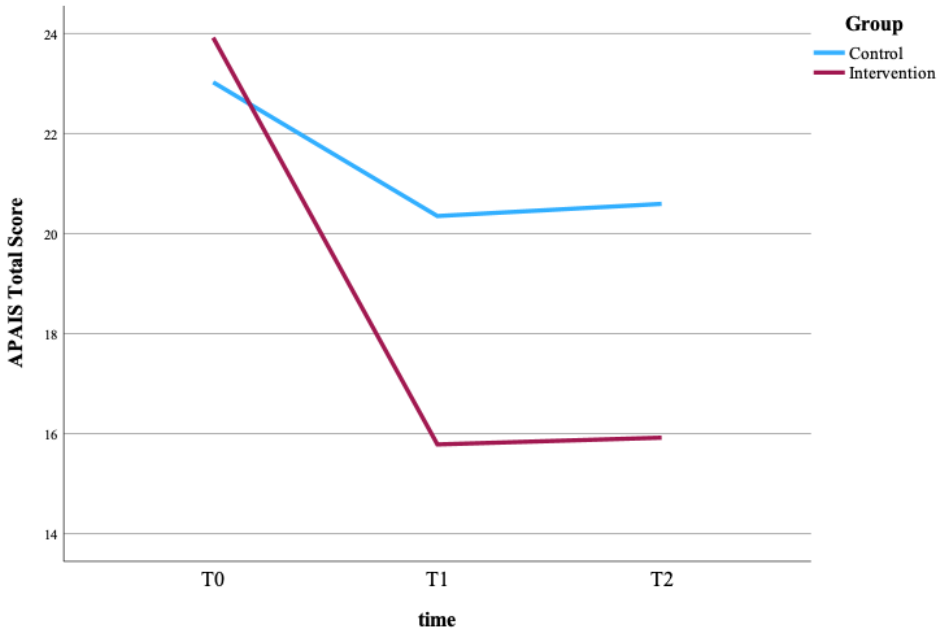

eFigure 2a. Changes in the preoperative anxiety scores across three measurement time points

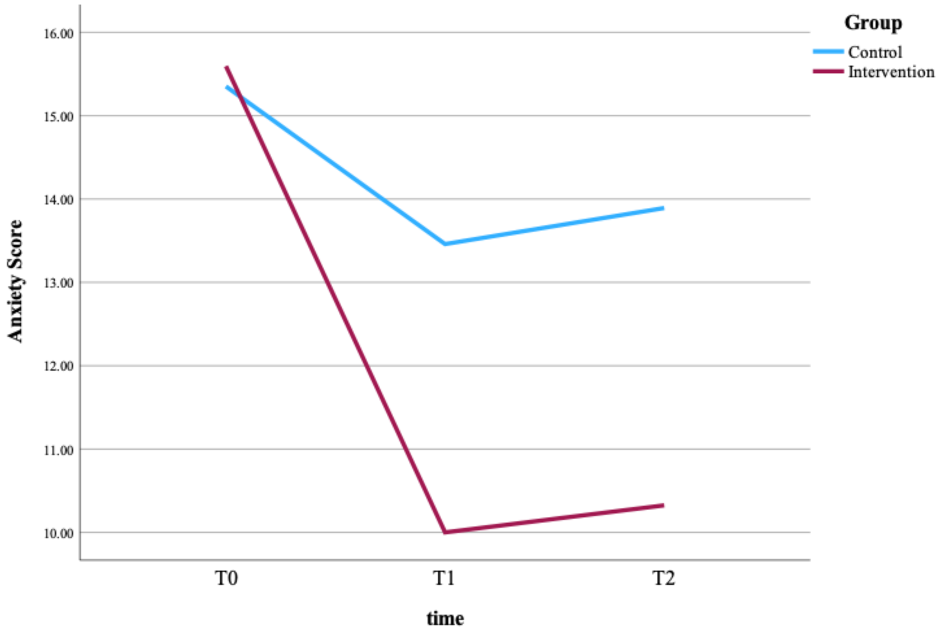

eFigure 2b. Changes of anxiety sub-scores across three time points

© 2023 Chiu PL et al. *JAMA Network Open.*

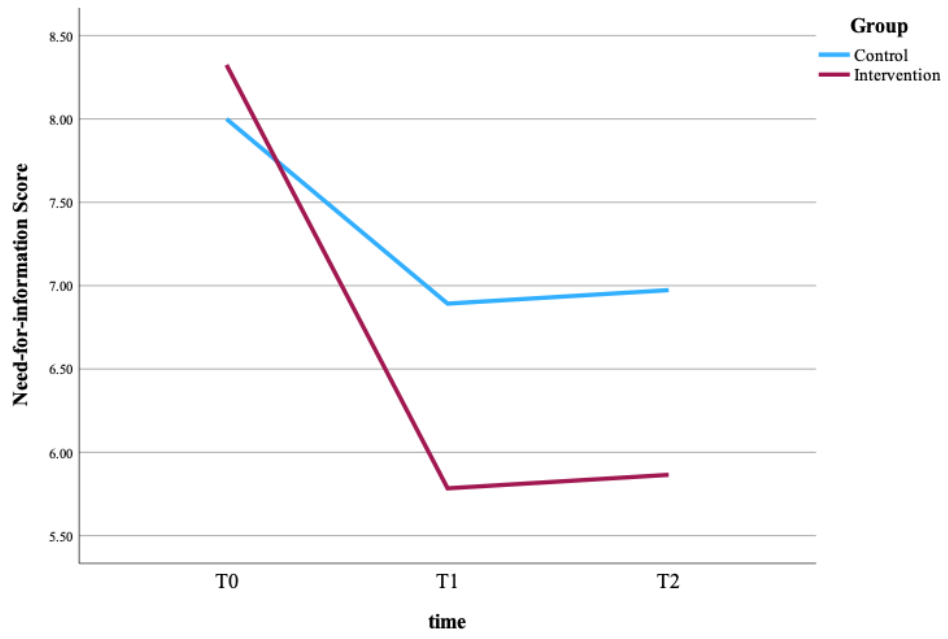

eFigure 2c. Changes of need-for-information sub-scores across three time points

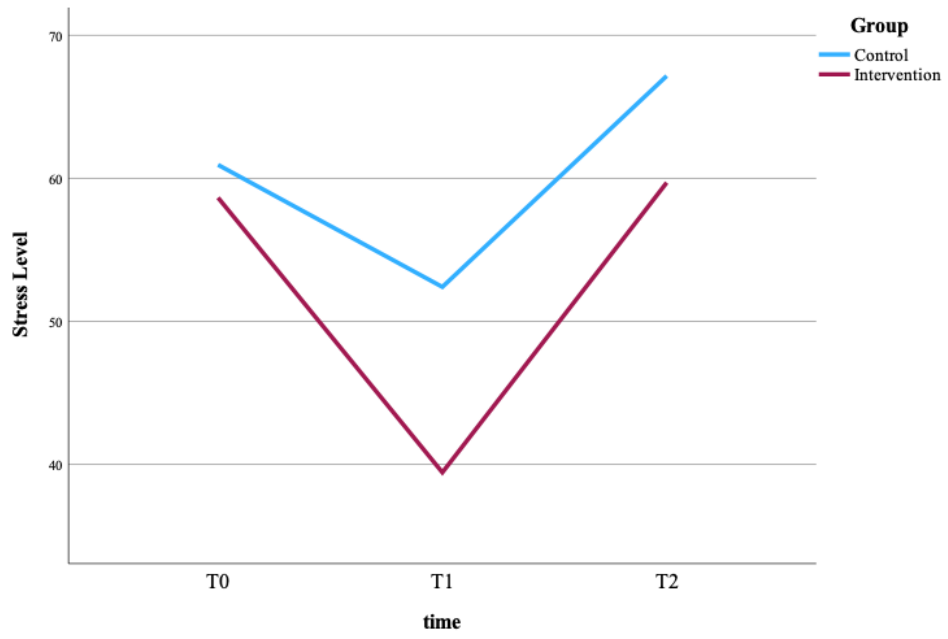

eFigure 3. Changes of Stress Level Across Three Time Points

© 2023 Chiu PL et al. *JAMA Network Open.*

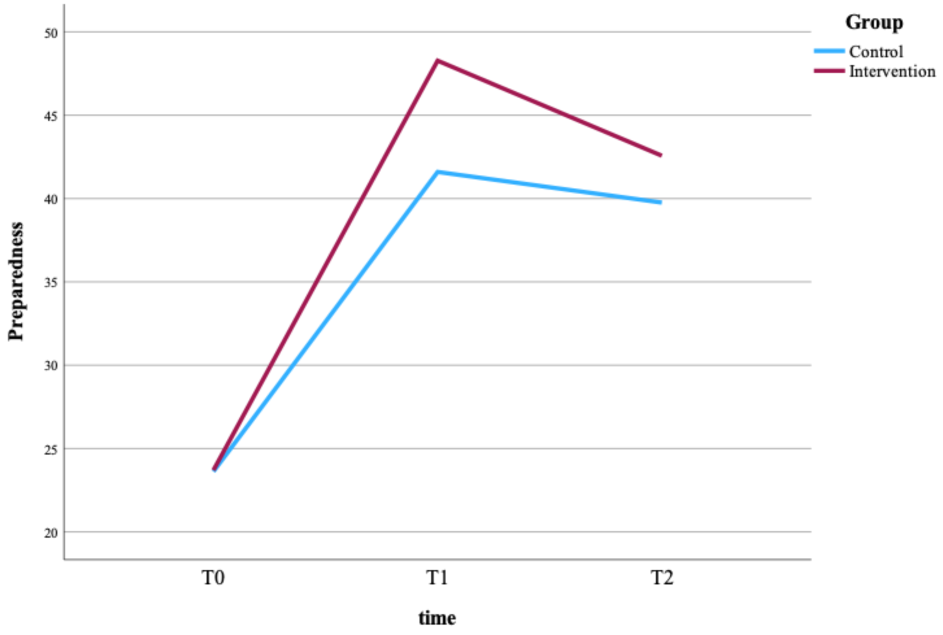

eFigure 4. Changes of Preparedness Across Three Time Points

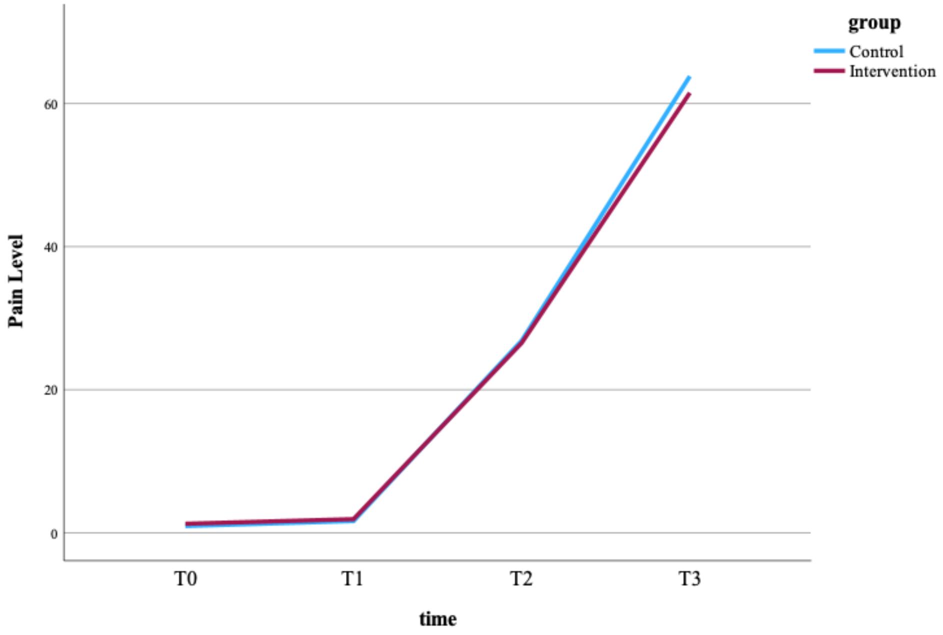

eFigure 5. Changes of Pain Level Across Four Time Points

© 2023 Chiu PL et al. *JAMA Network Open.*

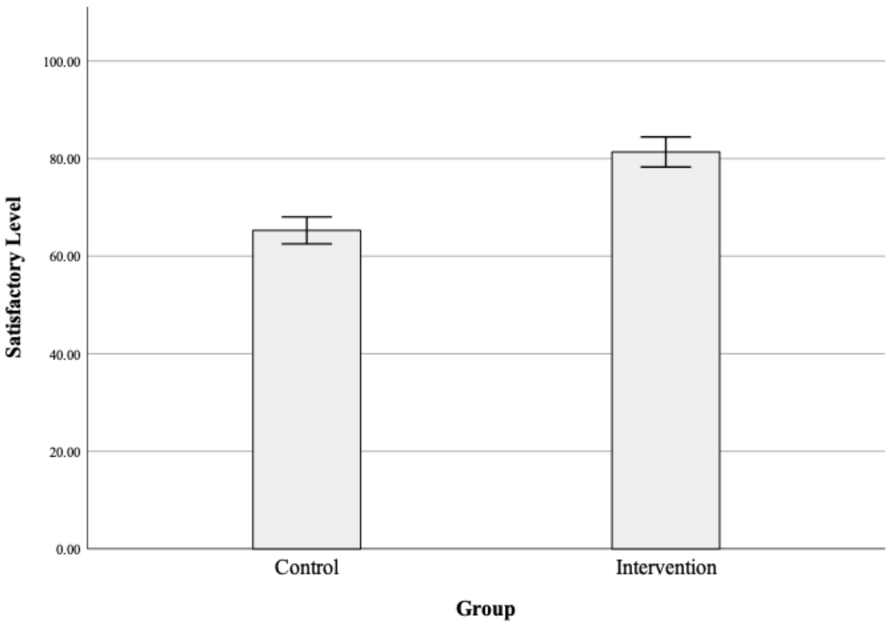

eFigure 6. Graphical Comparison of the Satisfaction of the Study Groups at T3

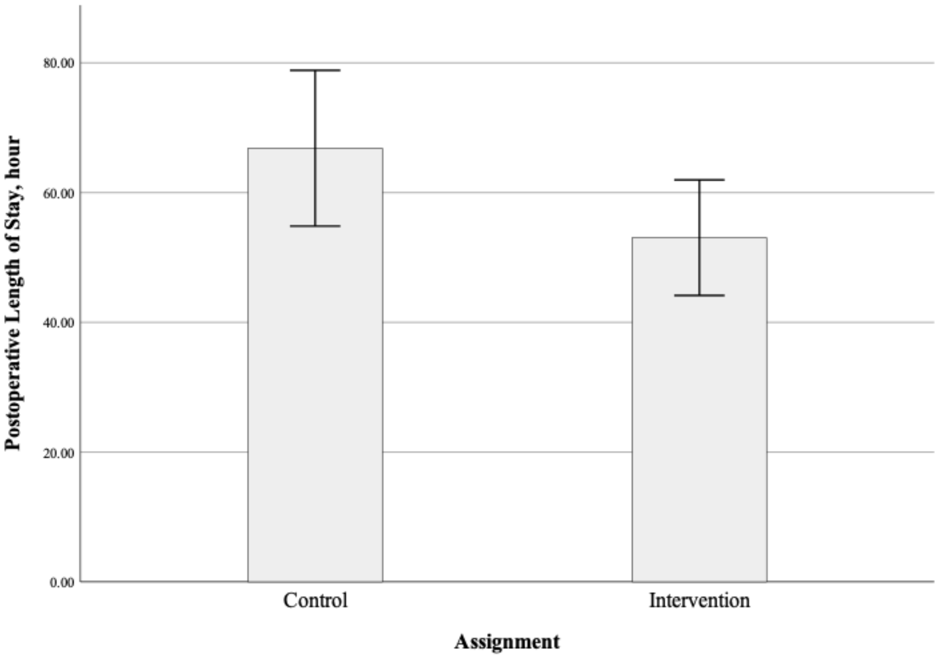

eFigure 7. Graphical Comparison of Postoperative Length of Stay Between Study Groups

© 2023 Chiu PL et al. *JAMA Network Open.*

eAppendix. A Detailed Description of Each Measure Used in This Trial

Primary outcome: Pre-operative anxiety

Pre-operative anxiety was measured using the Amsterdam Pre-operative Anxiety and Information Scale (APAIS).[1] The scale consists of six items divided into two subscales: the anxiety scale and the need-for-information scale. Higher scores indicate higher anxiety levels or greater information needs. Moerman et al. suggested that patients scoring 11 or higher on the anxiety scale should be considered anxiety cases. A previous study indicated that APAIS is a valid instrument for assessing pre-operative anxiety and is highly correlated with the state scale of the State-Trait Anxiety Inventory (STAI-S; > 0.60) and specific to pre-operative assessment.[2] This study used the translated and validated Chinese version of the APAIS,[3] with Cronbach's alphas of 0.862 and 0.830 for the anxiety and the need-for-information subscales, respectively.

Secondary outcomes: Pain

The visual analogue scale (VAS) was used to assess the subjective pain levels of the participants. It is a simple yet effective tool consisting of a continuous scale, a horizontal line 100 mm long anchored by two distinctive verbal descriptors of a symptom on a scale of 0 mm (none) to 100 mm (maximum). The VAS is quick to administer and widely adopted in similar studies[4-6] and is more sensitive for identifying fine changes than solely numerical scales and four-point scales, especially for assessing pain.[7]

Stress

The VAS was used to assess the participants' self-reported stress levels. The stress

VAS consisted of a 100mm horizontal line with the descriptors "none" at 0 mm and "maximum" at 100mm. Participants indicated their stress level by marking a vertical line along the horizontal scale.

Preparedness

Preparedness for surgery refers to the understanding of risks, benefits, alternatives, potential complications, and expected outcomes of upcoming surgery.[8] In this study, the operational definition of preparedness refers to the extent of the participants' feeling ready and equipped to undergo surgery.[9] The VAS was used to assess the subjects' preparedness on a scale of 0 mm (none) to 100 mm (maximum).

Simulation sickness

Simulation sickness refers to a syndrome similar to motion sickness that is often experienced during exposure to simulators or virtual reality (VR) environments.[10] In this study, the simulation sickness of the participants was assessed using the Simulation Sickness Questionnaire (SSQ) developed by Kennedy et al.[11] The SSQ is a self-report questionnaire comprising of 16 items related to simulator sickness. Respondents rate their level of discomfort or symptoms using a 4-point rating scale for each item: 0 (none), 1 (slightly), 2 (moderate) and 3 (severe). The items are grouped into three subscale clusters: Nausea (N), Oculomotor (O) and Disorientation (D), each reflecting the impact of that aspect of simulation sickness on the participant. A weighted score representing the Total Severity (TS) of simulation sickness experienced by the participant can be calculated. A higher score represents a greater level of simulation sickness. The SSQ has been adopted

© 2023 Chiu PL et al. *JAMA Network Open.*

by numerous studies on VR.[12] The Cronbach's alpha was 0.84 for nausea, 0.91 for oculomotor and 0.88 for discomfort.[11] The translated Chinese version[13,14] was used for data collection in this study after undergoing content validation by an expert panel.

Satisfaction

Satisfaction refers to the fulfilment or gratification of a desire, need or appetite.[15] In this study, the operational definition of satisfaction refers to the fulfilment or gratification of the participant by the pre-operative services. The self-report VAS was used to assess the participant's satisfaction on a scale of 0 mm (none) to 100 mm (maximum).

Postoperative length of stay

The length of stay refers to the duration of hospitalization between admission and discharge[16] and is an important metric for assessing the quality of care.[17] In this study, to minimize the confounding effects arising from varying pre-operative waiting times, the postoperative length of stay was operationally defined as the time from the end of surgery and anesthesia and transfer to the Post-Anesthetic Care Unit (PACU) to discharge from the surgical in-patient episode. Using the interval from the end of anesthesia and surgery, rather than initial hospital arrival or admission, provides a more standardized measure of the postoperative recovery time. The PACU admission marked the endpoint of the intraoperative period and the beginning of the postoperative recovery period. Defining the endpoint of the postoperative period as discharge from the surgical in-patient episode also provides a consistent measure across patients. The postoperative length of stay was measured in hours, rounded to the nearest whole number. The periods were calculated

based on data retrieved from the hospital's computerized health record systems, including the Operating Theatre Management System (OTMS) and Clinical Management System (CMS).

eReferences

1. Moerman N, van Dam FS, Muller MJ, Oosting H. The Amsterdam Pre-operative Anxiety and Information Scale (APAIS). *Anesth Analg.* 1996; 82(3); 445–451. doi:10.1097/00000539-199603000-00002
2. Sarah S, Lynn S. Is the Amsterdam Preoperative Anxiety and Information Scale (APAIS) a Valid Tool in Guiding the Management of Preoperative Anxiety in Adult Patients? A Literature Review. *J Nurs Pract*. 2019;3(1); 95–102. doi:10.36959/545/368
3. Wu H, Zhao X, Chu S, et al. Validation of the Chinese version of the Amsterdam Preoperative Anxiety and Information Scale (APAIS). *Health Qual Life Outcomes*. 2020;18(1):66. doi: 10.1186/s12955-020-01294-3
4. Bekelis K, Calnan D, Simmons N, MacKenzie TA, Kakoulides G. Effect of an Immersive Preoperative Virtual Reality Experience on Patient Reported Outcomes: A Randomized Controlled Trial. *Ann Surg*. 2017;265(6):1068-1073. doi: 10.1097/SLA.0000000000002094
5. Eijlers R, Dierckx B, Staals LM, et al. Virtual reality exposure before elective day care surgery to reduce anxiety and pain in children: A randomised controlled trial. *Eur J Anaesthesiol*. 2019;36(10):728-737. doi: 10.1097/EJA.0000000000001059.
6. Yang JH, Ryu JJ, Nam E, Lee HS, Lee JK. Effects of Preoperative Virtual Reality

© 2023 Chiu PL et al. *JAMA Network Open.*

Magnetic Resonance Imaging on Preoperative Anxiety in Patients Undergoing Arthroscopic Knee Surgery: A Randomized Controlled Study. *Arthroscopy*. 2019;35(8):2394-2399. doi: 10.1016/j.arthro.2019.02.037

7. Chiarotto A, Maxwell LJ, Ostelo RW, Boers M, Tugwell P, Terwee CB. Measurement Properties of Visual Analogue Scale, Numeric Rating Scale, and Pain Severity Subscale of the Brief Pain Inventory in Patients With Low Back Pain: A Systematic Review. *J Pain*. 2019;20(3):245-263. doi: 10.1016/j.jpain.2018.07.009
8. Greene KA, Wyman AM, Scott LA, Hart S, Hoyte L, Bassaly R. Evaluation of patient preparedness for surgery: a randomized controlled trial. *Am J Obstet Gynecol*. 2017;217(2):179.e1-179.e7. doi: 10.1016/j.ajog.2017.04.017
9. Kenton K, Pham T, Mueller E, Brubaker L. Patient preparedness: an important predictor of surgical outcome. *Am J Obstet Gynecol*. 2007;197(6):654.e1-6. doi: 10.1016/j.ajog.2007.08.059
10. Dużmańska N, Strojny P, Strojny A. Can Simulator Sickness Be Avoided? A Review on Temporal Aspects of Simulator Sickness. *Front Psychol*. 2018;9:2132. doi: 10.3389/fpsyg.2018.02132
11. Kennedy RS, Lane NE, Berbaum KS, Lilienthal MG. (1993). Simulator Sickness Questionnaire: An Enhanced Method for Quantifying Simulator Sickness. *Int J Aviat Psychol*. 1993;3(3); 203–220. oi:10.1207/s15327108ijap0303_3
12. Bimberg P, Weissker T, Kulik A. On the usage of the simulator sickness questionnaire for virtual reality research. *2020 IEEE conference on virtual reality and 3D user interfaces abstracts and workshops (VRW). IEEE*. 2020; 464-467.

13. Chen W, Chen J Z, So R H Y. Visually induced motion sickness: Effects of translational visual motion along different axes. *Contemp Ergon Hum Factors*. 2011; 281-287.

14. Ng TY. *Sense-of-presence in virtual and real environments showing similar content: Questionnaire development and relationships among sense-of-presence, performance and cybersickness*. Hong Kong University of Science and Technology, 2002.

15. Pascoe GC. Patient satisfaction in primary health care: A literature review and analysis. Eval Program Plann. 1983; 6(3–4); 185–210. doi:10.1016/0149-7189(83)90002-2

16. Williams RL. Survey Sampling and Weighting. *Encyclopedia Health Econ*. 2014; 371–374. doi:10.1016/B978-0-12-375678-7.00703-3

17. Carter EM, Potts HW. Predicting length of stay from an electronic patient record system: a primary total knee replacement example. *BMC Med Inform Decis Mak*. 2014;14:26. doi: 10.1186/1472-6947-14-26

© 2023 Chiu PL et al. *JAMA Network Open.*
