# Supplementary material for: Virtual Reality–Based Intervention to Reduce Preoperative Anxiety in Adults Undergoing Elective Surgery: A Randomized Clinical Trial
Source: JAMA Netw Open. 2023 Oct 31;6(10):e2340588. doi: 10.1001/jamanetworkopen.2023.40588 (PMC10618840; doi:10.1001/jamanetworkopen.2023.40588)
Supplement: Supplement 3. — Data Sharing Statement [file jamanetwopen-e2340588-s003.pdf]

Data Sharing Statement

Chiu. Virtual Reality–Based Intervention to Reduce Preoperative Anxiety in Adults Undergoing Elective Surgery. *JAMA Netw Open*. Published October 31, 2023.
doi:10.1001/jamanetworkopen.2023.40588

Data
Data available: No

Additional Information
Explanation for why data not available: The datasets generated and analysed during the current study are not publicly available due to privacy protection and ethical considerations but are available from the corresponding author on reasonable request.
